# Whole Blood Gene Expression Profile Associated with Spontaneous Preterm Birth in Women with Threatened Preterm Labor

**DOI:** 10.1371/journal.pone.0096901

**Published:** 2014-05-14

**Authors:** Yujing Jan Heng, Craig Edward Pennell, Hon Nian Chua, Jonathan Edward Perkins, Stephen James Lye

**Affiliations:** 1 Lunenfeld-Tanenbaum Research Institute, Mount Sinai Hospital, Toronto, Ontario, Canada; 2 School of Women's and Infants' Health, University of Western Australia, Crawley, WA, Australia; 3 Institute for Infocomm Research, Agency for Science, Technology and Research, Singapore, Singapore; Fudan University, China

## Abstract

Threatened preterm labor (TPTL) is defined as persistent premature uterine contractions between 20 and 37 weeks of gestation and is the most common condition that requires hospitalization during pregnancy. Most of these TPTL women continue their pregnancies to term while only an estimated 5% will deliver a premature baby within ten days. The aim of this work was to study differential whole blood gene expression associated with spontaneous preterm birth (sPTB) within 48 hours of hospital admission. Peripheral blood was collected at point of hospital admission from 154 women with TPTL before any medical treatment. Microarrays were utilized to investigate differential whole blood gene expression between TPTL women who did (n = 48) or did not have a sPTB (n = 106) within 48 hours of admission. Total leukocyte and neutrophil counts were significantly higher (35% and 41% respectively) in women who had sPTB than women who did not deliver within 48 hours (*p*<0.001). Fetal fibronectin (fFN) test was performed on 62 women. There was no difference in the urine, vaginal and placental microbiology and histopathology reports between the two groups of women. There were 469 significant differentially expressed genes (FDR<0.05); 28 differentially expressed genes were chosen for microarray validation using qRT-PCR and 20 out of 28 genes were successfully validated (*p*<0.05). An optimal random forest classifier model to predict sPTB was achieved using the top nine differentially expressed genes coupled with peripheral clinical blood data (sensitivity 70.8%, specificity 75.5%). These differentially expressed genes may further elucidate the underlying mechanisms of sPTB and pave the way for future systems biology studies to predict sPTB.

## Introduction

Preterm birth (PTB; birth at <37 weeks of gestation) occurs in about 8–11% of pregnancies worldwide and remains the main cause of perinatal mortality and morbidity in the developed world [1]. Medical advances have increased the survival rates of premature babies; however, premature infants remain vulnerable to disabilities such as respiratory disorders, cognitive impairment, blindness, deafness etc. [2]. In later life, they may face complications such as motor and sensory impairment, learning difficulties and behavioral issues. Prematurity leads to an immediate and long term emotional and financial burden to families, communities and the health care system [3]–[7].

Threatened preterm labor (TPTL) is defined as persistent premature uterine contractions between 20 and 37 weeks of gestation and may include other symptoms such as pelvic pressure, backache, increased vaginal discharge, menstrual-like cramps, bleeding/show and shortened cervix [8]–[11]. Treatment of TPTL involves administration of tocolytic agents to temporarily inhibit uterine contractions and prolong the pregnancy up to 48 hours. This 48 hour window serves to achieve both the benefits of corticosteroid administration on fetal lung maturation as well as the ability to transport the woman to a tertiary hospital with advanced neonatal facilities [12], [13].

Generally, labor contractions in the majority of TPTL women will cease and they often continue their pregnancies to term [14]; while approximately 5% of these women will progress into true PTL and deliver a premature baby within 10 days [15]. Thus, women in “false labor” are subjected to unnecessary hospitalization, medical intervention, psychologic stress, exposed to drug side effects and contribute to healthcare costs [16], [17]. For example, although corticosteroids augment fetal lung maturity and reduce respiratory distress syndrome, intraventricular hemorrhage and neonatal mortality in premature infants [18], their longer term side effects remain unclear [19]–[22].

There is limited success for clinicians to accurately stratify TPTL women into “true PTL” or “false labor” at the point of hospital admission. Currently, predicting PTB involves the assessment of clinical risk factors [23], detecting a short cervix (<15 mm) [1], [24] and the presence of fetal fibronectin (fFN) in the cervicovaginal fluid [8], [25]. fFN testing is clinically useful for its high negative predictive value (NPV) [15]. However, fFN cannot be performed on women who had prior vaginal/cervical examination, unprotected sexual intercourse, antepartum hemorrhage or ruptured fetal membranes as these factors can influence the fFN test results [26]. As a result, about 50% of TPTL women are generally ineligible for fFN testing.

The ability to predict PTB at point of hospital admission would provide clinicians the opportunity to focus therapeutic interventions on women who are more likely to deliver within the next 48 hours, whilst women in “false labor” can be offered supportive care and discharged. Therefore, a new diagnostic test that predicts PTB, using an easily accessible biological fluid (e.g. blood) and can be performed on all TPTL patients will be beneficial. This capability will allow a more rational approach to manage TPTL and provide considerable cost savings to the healthcare system. Recently, Chim and colleagues utilized gene expression microarray to identify placental genes associated with spontaneous PTB (sPTB) and subsequently screened for placental RNA transcripts and microRNAs in the maternal plasma in an attempt to discover novel biomarkers associated with sPTB [27].

Hence, this study utilized microarrays to characterize whole blood gene expression in women with TPTL. The specific hypotheses were: 1) women in TPTL who progress to true PTL and had a sPTB within 48 hours have a different gene expression profile compared with women who did not deliver within 48 hours, and 2) a genomic expression signature can predict sPTB within 48 hours in TPTL women. We obtained a nine gene signature coupled with peripheral clinical blood data to predict sPTB with 70.8% sensitivity and 75.5% specificity. Differentially expressed genes and their respective proteins may further elucidate the underlying mechanisms of sPTB and pave the way for the development of more precise and targeted therapies.

## Methods and Materials

### Patient recruitment and ethics statement

The study was approved by the Ethics Committee, Department of Health, Government of Western Australia (EC05-34.3). Participants provided written consent; consent procedure was also approved by the Ethics Committee. The majority of participants were of Caucasian ethnicity. Using a two class comparison model (with restrictive inclusion and exclusion criteria), an alpha of 0.001, beta of 0.8, four technical replicates and estimating the gene expression variance in human biological samples to be 0.5, the minimum sample size to identify a two-fold change in gene expression is 26 patients (biological replicates) with 13 in each group. Given that the proportion of patients who will progress to preterm delivery is somewhat variable, 300 women admitted with TPTL were recruited from King Edward Memorial Hospital, Perth, WA, Australia. Inclusion criteria at recruitment were presentation between 24 to 36 weeks' gestation, uterine contractions, with or without cervical changes, if dilated, cervix must be <4 cm, intact fetal membranes and no evidence of clinical chorioamnionitis (febrile >37.5°C, uterine tenderness, mother systemically unwell, fetal tachycardia). Exclusion criteria were as follows: excessive antepartum hemorrhage, preterm prelabor rupture of membranes, clinical chorioamnionitis, fetal anomaly, preeclampsia, intra-uterine growth restriction, diabetes mellitus or gestational diabetes or multi-fetal pregnancy.

fFN test (QuikCheck fFN, Hologic, Inc., Marlborough, MA) was performed where feasible (i.e. intact fetal membranes, no unprotected sexual intercourse in the preceding 48 hours, no prior vaginal examination and no bleeding) as part of the hospital's protocol and for comparison with microarray data. High vaginal swabs and urine were collected from each participant for standard hospital bacteria vaginosis screening, microscopy, culture and sensitivities as part of their routine clinical care. Women who delivered preterm at our hospital had their placentae sent for microscopy, culture and formal histopathology. Clinical microbiology assessment of vagina, urine and placenta cultures and placenta histopathology were reported where available. The lack of placenta reports in women who did not deliver within 48 hours was either due to them subsequently delivering at another hospital or histology was not routinely performed on term placentas. Participants were subsequently stratified into two groups: sPTB within 48 hours of hospital admission and no sPTB within 48 hours.

### Blood sample collection, RNA extraction and quality check

Maternal blood samples were collected at point of hospital admission prior to any medical treatment. Five PAXgene blood collection tubes (PreAnalytics, Hombrechtikon, Switzerland) were collected from each participant, total RNA was isolated using PAXgene Blood RNA system kit (QIAGEN, Doncaster, Victoria, Australia). RNA quality check was done at The Centre for Applied Genomics (TCAG; The Hospital for Sick Children (SickKids), Toronto, ON, Canada) using an Agilent 2100 BioAnalyser with the RNA 6000 Nano Kit (Agilent Technologies, Santa Clara, CA). The bioanalyser provides a RNA integrity number (RIN) to gauge RNA integrity, compare samples and ensure the repeatability of experiments. RIN is calculated using an algorithm and the bioanalyzer's electrophoretic trace where a RIN score of one represents strongly degraded RNA and a score of 10 represents intact RNA [28]. Adhering to TCAG's protocol, microarrays were only performed on samples with RIN greater than six.

### Microarray

Full microarray experiments were performed by TCAG. Total RNA samples were hybridized to Affymetrix Human Genome U133 Plus 2.0 arrays (Affymetrix, Santa Clara, CA). The globin reduction protocol was incorporated into microarray processing [29]. Microarray data have been deposited in NCBI's Gene Expression Omnibus and are accessible through GEO Series accession number GSE46510:

(http://www.ncbi.nlm.nih.gov/geo/query/acc.cgi?acc=GSE46510).

Clinical data associated with the microarray data are presented in Table S1 in File S1.

### Differential gene expression analysis

Affymetrix U133 Plus2 GeneChip CEL files were normalized using the GeneChip Robust Multiarray Average (GCRMA) (Bioconductor, R). Custom (Gene)Chip Definition Files (CDFs) for Entrez Gene (version 17) were used to map Affymetrix GeneChip probes to transcripts/exons/genes for specific databases [30]. Differential gene expressions were analyzed using *Limma* (Bioconductor, R) [31]. To correct for multiple hypotheses testing, significant differentially expressed genes were identified based on a false discovery rate (FDR) threshold of <0.05 using the Benjamini and Hochberg approach. Fold changes were calculated using median values and expressed as logarithm base 2 (Log_2_).

Gene Ontology (GO) Slim annotations were obtained for significant genes (*Limma* FDR<0.05) [32], [33]. Reactome Functional Interaction Cytoscape plug-in (version 4.0 beta) was used to visualize community networks and to determine if differently expressed genes (passing *Limma* FDR threshold of <0.05) may be enriched and form clusters of interconnected molecular events or ‘reactions’ associated with sPTB within 48 hours [34], [35]. Representative GO Slim terms were obtained for each cluster. Enrichment analyses using FuncAssociate 2.0 were performed separately on genes passing *Limma* FDR threshold of <0.1 that were either up or down regulated [36]; and pathway analyses were performed using DAVID Bioinformatics Resources 6.7 (BioCarta, KEGG and Panther) with genes passing *Limma* FDR threshold of <0.1 [37]. For both enrichment and pathway analyses, the total number of genes observed in the microarray (n = 19,008) was used as the background. A less stringent FDR of <0.1 allows more genes to be included in the analyses.

### Quantitative real time-PCR

Quantitative real-time PCR (qRT-PCR) was performed to validate a subset of significant genes that displayed a Log_2_ fold change of ±0.6 (i.e. ≥50% increase or ≥34% decrease in women who delivered within 48 hours) and a minimum average microarray intensity expression of >4 (CEL files arbitrary expression values) [38], [39]. Primers were designed using Primer BLAST. Primer specificities and efficiencies (65%–120%) were determined using pooled cDNA from 10 women (five term deliveries, five preterm deliveries) and five-point standard curves.

Reverse transcription was performed to obtain 50 ng/µL cDNA using iScript (BIO-RAD, Hercules, CA): 25°C for 5 min, 42°C for 30 min, 85°C for 5 min. qRT-PCR was performed in triplicates using LuminoCt SYBR Green PCR ReadyMix (Sigma-Aldrich, St. Louis, MO): 90°C for 30 s, 95°C for 5 s, 60°C for 20 s (40 cycles) on the CFX384 Touch Real-Time PCR Detection System (BIO-RAD). qRT-PCR expression data were normalized to three optimized housekeeping genes (*TBP, SDHA* and *YWHAZ*; average expression stability determined with geNorm was M = 0.131 [40]) and corrected for primer efficiencies using CFX Manager 2.0 (BIO-RAD). Fold changes were calculated using median values.

### Statistical analysis

Demographics, peripheral clinical blood data and microbiology data comparison between women who did or did not have a sPTB within 48 hours were done using the Mann-Whitney *U* test and Fisher's exact test; correlation was performed using Pearson's *r* (Statistical Package for Social Sciences version 17.0, SPSS Inc, Chicago, IL). qRT-PCR data analyses were performed using binary logistic regression (LogXact 10, Cytel Inc, Cambridge, MA). The dot plots were produced using GraphPad Prism v5.02 (GraphPad Software, San Diego, CA).

### Random forest classifier

A random forest classifier model was constructed to differentiate between women who had a sPTB within 48 hours and those who did not. The dataset consists only of numeric values and missing values were imputed with the median across the samples. The model was built using the random forest implementation (*randomForest* version 4.6-7, R). Leave-one-out cross validation was performed to provide out-of-bag evaluation of the classifier's performance. Receiver Operating Characteristics (ROC) curve was used to assess prediction performance.

## Results

**Figure 1:**
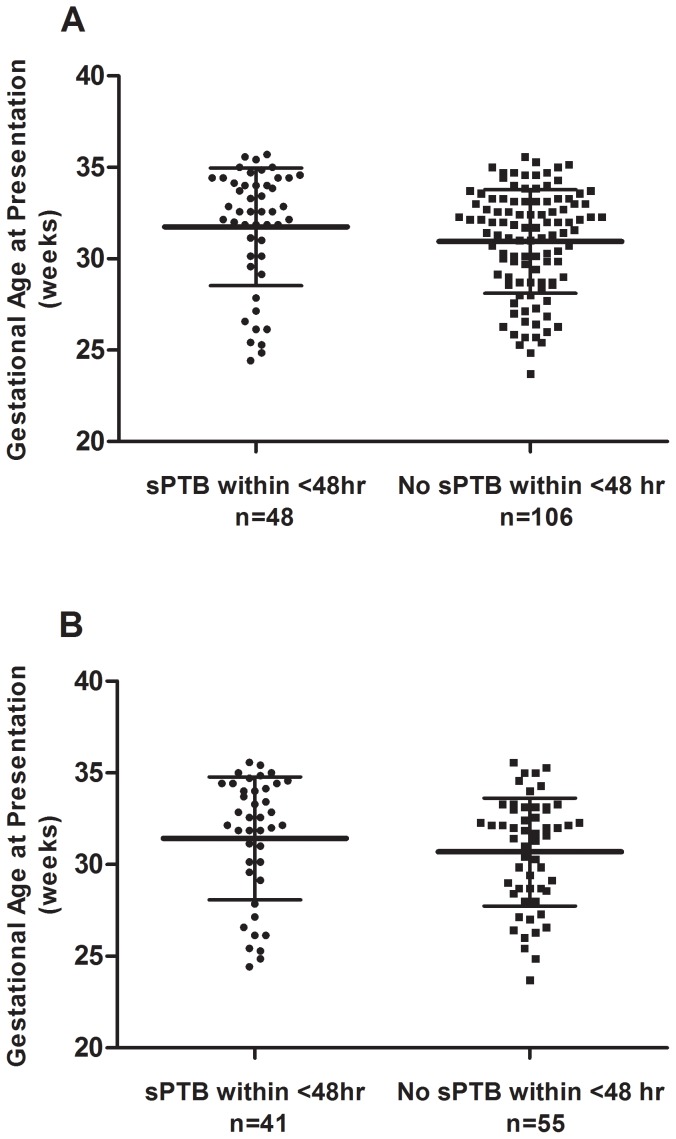
The dot plots (mean and standard deviations) of gestational age at presentation of women who did or did not have a spontaneous preterm birth (sPTB) within 48 hours of hospital admission in the microarray (A) and qRT-PCR (B) study, respectively.

Two hundred out of 300 samples were retrospectively selected based on delivery outcome and re-examined for eligibility adhering to our inclusion and exclusion criteria; 178 samples were confirmed for eligibility and sent to the microarray facility. Twenty four samples were unfavorable post RNA quality check or post microarray procedure (chip defect/error) thus resulting in 154 samples for final data analysis (Table 1). There were 48 women who had a sPTB within 48 hours of hospital admission and 106 women who did not. Gestational age at presentation was significantly different between women who had or did not have a sPTB within 48 hours. Figure 1 displays the gestational age at presentation of women in the microarray and qRT-PCR studies, respectively. Peripheral blood clinical laboratory results were obtained from 125 women. Total leukocyte and neutrophil counts were 34.7% and 40.8% higher in women who had sPTB within 48 hours of hospital admission (*p*<0.001). There were no differences in urine culture, vaginal microbiology as well as placental histopathology and microbiology assessment between the two groups (*p*>0.05; Table 2). No woman had bacteria vaginosis. fFN test was performed on 62 patients out of 154 patients (40.3%), with 83.3% sensitivity, 66.0% specificity, 37.0% and 94.3% positive and negative predictive values (PPV and NPV) respectively.

**Table 1:** Clinical demographics of the 154 study participants.

	Spontaneous preterm birth within 48 hours	No spontaneous preterm birth within 48 hours				Mann-Whitney *U* Test (*p*-value)
		Total	Preterm birth after 48 hours and within 7 days	Preterm birth at >7 days and <37 weeks	Term Delivery	
***n***	48	106	12	15	79	
**Maternal Age** (mean years ±SD)	28.2±5.8	28.3±5.8	27.4±7.9	28.0±5.3	28.5±5.7	0.827
**Gestational Age at Presentation** (mean weeks±SD)	31.7±3.2	30.9±2.8	28.8±3.0	31.4±1.4	31.2±2.9	0.044*
**Gravidity** (mean±SD)	2±2	3±2	2±1	4±2	3±2	
**Parity** (mean±SD)	1±1	2±2	1±1	2±1	2±2	
**Cervical Dilatation at Presentation** (mean cm±SD)	2±1	1±1	2±1	1±1	1±1	
**Gestational Age at Delivery** (mean weeks±SD)	31.8±3.3	37.4±3.6	29.5±3.1	34.8±2.1	39.0±1.2	<0.001*
**Leukocyte Counts∧** (mean 10^9^/L±SD)	16.3±4.9	12.1±3.1	13.9±3.8	11.8±3.2	11.8±2.8	<0.001*
**Neutrophil Counts∧** (mean 10^9^/L±SD)	13.8±5.1	9.8±3.1	11.6±4	9.6±2.9	9.5±2.8	<0.001*
**Lymphocyte Counts∧** (mean 10^9^/L±SD)	6±28.8	1.6±0.7	1.6±0.5	1.6±0.8	1.6±0.8	0.204
**Monocyte Counts∧** (mean 10^9^/L±SD)	0.6±0.4	0.6±0.3	0.6±0.3	0.5±0.3	0.6±0.3	0.803
**Hemoglobin∧** (mean grams/L±SD)	122±15	119±10	123±12	123±13	118±10	0.275
**Previous Preterm Birth (** ***n*** **)**	7/48 (14.6%)	25/106 (23.6%)	5/12 (41.7%)	7/15 (46.7%)	13/79 (16.5%)	
**Smoker (** ***n*** **)**	14/48 (29.2%)	29/106 (27.4%)	4/12 (33.3%)	3/15 (20.0%)	22/79 (27.8%)	
**Fetal Fibronectin Performance**						
**Total Tests (** ***n*** **)**	12/48 (25.0%)	50/106 (47.2%)	4/12 (33.3%)	9/15 (60.0%)	38/79 (48.1%)	
**Positive (n)**	10	17	3	6	8	
**Negative (n)**	2	33	0	3	30	

∧Peripheral blood data (total leukocytes, neutrophils, lymphocytes, monocytes counts and hemoglobin) were obtained from 125 women (<48 hours delivery n = 45, >48 hours delivery n = 80). *Significantly different between preterm delivery within 48 hours (n = 48) and no preterm delivery within 48 hours (n = 106).

**Table 2:** Urine culture, vaginal microbiology and placental histopathology and microbiologic assessments of participants.

A. Urine and vaginal microbiology	Spontaneous preterm birth within 48 hours (n = 48)	No preterm birth within 48 hours (n = 106)	Fisher's test *p*-value
**Urine Culture (total ** ***n*** ** with reports)**	**26**	**83**	**0.852**
No growth	15	40	
Mixed growth	9	33	
Pathogen isolated *Escherichia coli* positive	1	2	
* Bacillus cereus* positive		1	
Group B streptococcus (GBS) and *Staphylococcusaureus* positive		1	
Contamination reported	1	6	
No report available	22	23	
**Vaginal Culture (total ** ***n*** ** with reports)**	**29**	**77**	**0.189**
No growth	3	3	
Normal vaginal flora	6	31	
*Ureaplasma* spp. positive	4	14	
GBS positive	3	7	
*Candida* spp. positive	3	9	
Other pathogens isolated			
* Staphylococcus aureus* positive		1	
* Escherichia coli* or coliform bacteria positive	2		
Co-presence of ≥2 microorganisms (*Candida* spp., *Ureaplasma* spp., GBS, *Mycoplasma* spp., *Neisseria* spp., *Gardnerella* spp., *Haemophilus* spp., unspecified coliform)	8	12	
No report available	19	29	

B Placental histopathology and microbiologic assessments	Spontaneous preterm birth within 48 hours (n = 48)	No preterm birth within 48 hours (n = 106)	Fisher's test *p*-value
**Histology consistent with immature placenta (total ** ***n*** **)**	**10**	**4**	**0.578**
No growth	4	4	
Mixed flora	1		
GBS positive	2		
Microbiology not performed	3		
**Chorioamnionitis (mild to severe) (total ** ***n*** **)**	**10**	**8**	
No growth	3	4	
Mixed flora	2		
GBS or *Escherichia coli* or *Ureaplasma* spp. positive	2	3	
Microbiology not performed	3	1	
**Decidual vasculopathy (total ** ***n*** **)**	**2**	**1**	
*Ureaplasma* spp. or *Mycoplasma hominis* or GBS positive	2	1	
**Ischaemia (total ** ***n*** **)**		**1**	
Microbiology not performed		1	
**No histopathology report (total ** ***n*** **)**	**26**	**92**	

### Identification of differentially expressed genes using microarray

**Figure 2:**
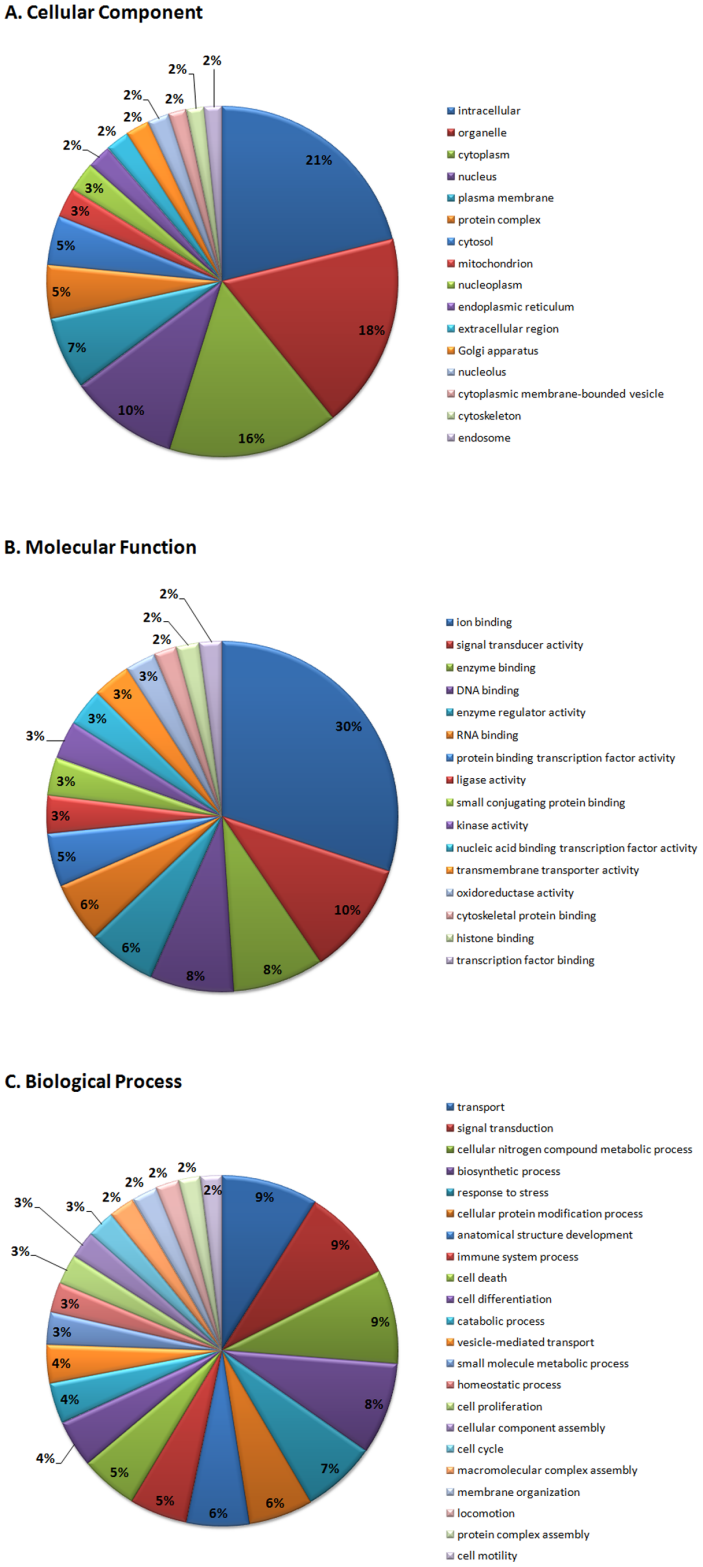
GO Slim terms of (A) cellular components, (B) molecular functions and (C) biological process that are representative of the 469 significantly expressed genes.

As gestational age at presentation was significantly different between women who did or did not have a sPTB within 48 hours, this suggested that gene expression in whole blood may be gestational age dependent. We thus analyzed the microarray data by pairing matched gestational age samples using a modified *Limma* design matrix (moderated paired *t*-test). Samples were rounded down to the nearest gestational week and then grouped according to their gestational age at presentation. Data analysis using *Limma* obtained 469 significantly differentially expressed genes (256 up-regulated genes, 213 down-regulated genes, FDR<0.05; Table S2 in File S1). Table 3 displays the top 20 up and down regulated genes (ranked by Log_2_ fold change). Of the 469 significant genes, 176 genes were confidently mapped to relevant GO Slim terms, 254 genes were reported as ambiguous and 39 genes were not annotated [32] (Figure 2).

**Table 3:** Top twenty significantly expressed genes (FDR<0.05, *Limma*) ranked by fold change.

Gene	Fold change (Log2)	Fold change	Preterm birth within 48 hours Median expression (25^th^, 75^th^ percentile)	No preterm birth within 48 hours Median expression (25^th^, 75^th^ percentile)	FDR	Gene annotation
**Up-regulated genes (n = 12)**						
ZDHHC19	1.202	2.30	4.66 (3.95, 5.51)	3.47 (3.19, 4.21)	0.000	zinc finger, DHHC-type containing 19
HPGD	1.049	2.07	5.80 (5.11, 7.60)	5.08 (4.17, 6.95)	0.047	hydroxyprostaglandin dehydrogenase 15-(NAD)
GPR84	0.990	1.99	8.00 (6.77, 8.78)	6.78 (6.40, 7.64)	0.007	G protein-coupled receptor 84
OPLAH	0.934	1.91	6.62 (5.89, 7.90)	5.82 (5.26, 6.69)	0.003	5-oxoprolinase (ATP-hydrolysing)
TDRD9	0.906	1.87	4.86 (3.68, 6.08)	3.89 (3.24, 4.94)	0.018	tudor domain containing 9
ATP9A	0.880	1.84	7.86 (6.73, 8.92)	6.71 (6.15, 7.78)	0.013	ATPase, class II, type 9A
GALNT14	0.879	1.84	9.04 (8.46, 9.58)	8.27 (7.86, 8.66)	0.000	UDP-N-acetyl-alpha-D-galactosamine:polypeptide N-acety lgalactosaminyltransferase 14
SLC26A8	0.802	1.74	7.53 (6.71, 8.08)	6.76 (6.05, 7.48)	0.015	solute carrier family 26, member 8
CDK5RAP2	0.768	1.70	8.73 (7.73, 10.10)	8.12 (7.44, 8.80)	0.017	CDK5 regulatory subunit associated protein 2
ST3GAL4-AS1	0.760	1.69	9.15 (8.41, 9.81)	8.41 (7.71, 9.03)	0.015	ST3GAL4 antisense RNA 1 (head to head)
G0S2	0.758	1.69	7.56 (7.10, 8.05)	6.83 (6.26, 7.53)	0.006	G0/G1switch 2
SOCS3	0.757	1.69	8.45 (7.51, 9.13)	7.77 (7.14, 8.22)	0.003	suppressor of cytokine signaling 3
**Down-regulated genes (n = 8)**						
METTL18	−0.909	0.53	3.46 (2.73, 3.79)	4.20 (3.52, 5.00)	0.002	methyltransferase like 18
GOLGA8A	−0.874	0.55	8.48 (7.63, 9.36)	9.32 (8.40, 10.26)	0.037	golgin A8 family, member A
ZNF302	−0.802	0.57	3.20 (2.76, 3.53)	3.51 (3.12, 4.37)	0.007	zinc finger protein 302
TRMT13	−0.797	0.58	4.10 (3.53, 5.12)	5.19 (3.74, 6.15)	0.047	tRNA methyltransferase 13 homolog (S. cerevisiae)
CETN3	−0.773	0.59	4.20 (3.01, 4.36)	4.41 (3.98, 5.70)	0.032	centrin, EF-hand protein, 3
ZFAND1	−0.771	0.59	5.35 (4.68, 6.31)	6.18 (5.12, 6.99)	0.040	zinc finger, AN1-type domain 1
STAP1	−0.764	0.59	4.10 (3.52, 4.52)	4.78 (4.09, 5.50)	0.007	signal transducing adaptor family member 1
MS4A1	−0.758	0.59	6.93 (6.09, 7.55)	7.76 (6.97, 8.69)	0.028	membrane-spanning 4-domains, subfamily A, member 1

### Enrichment analyses

**Figure 3:**
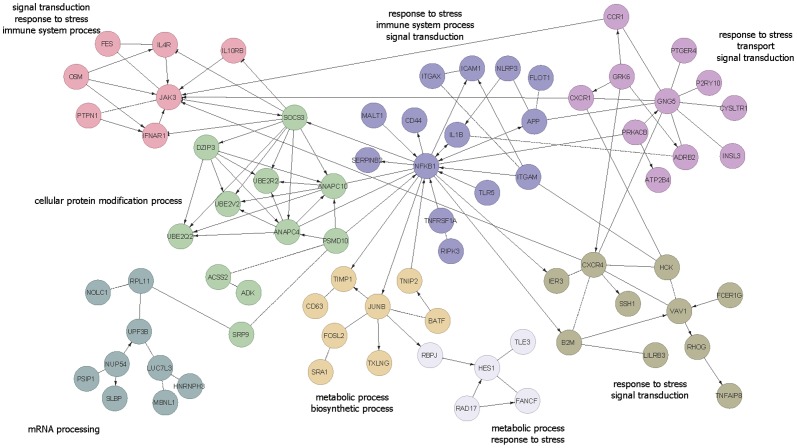
Reactome Functional Interaction analysis of 469 significant genes revealed eight clusters consisting of at least five of genes. Each cluster is indicated by a different color and their representative GO slim term(s). Edges of “->” indicate activating/catalyzing; “-|” for inhibition; “-” for functional interactions extracted from complexes or inputs and “- - -” for predicted functional interactions.

From the Reactome network constructed around the 469 differentially expressed genes (*Limma* FDR<0.05), eight major clusters with minimum five genes were discovered. These clusters generally represent metabolic process, response to stress, immune system process and signal transduction (Figure 3). A total of 1333 genes (*Limma* FDR<0.1) were subjected to functional enrichment analysis. Enrichment analyses found 14 functions (odds ratio >2) significantly enriched in 570 up-regulated genes and 17 functions (odds ratio >2) significantly enriched in 763 down-regulated genes (Table 4). No significant pathway analysis was obtained using DAVID.

**Table 4:** Functional enrichment analysis of a total of 1333 genes (FDR<0.1, *Limma*) resulted in 31 enriched GO terms with odds ratio of >2 (adjusted *p*<0.05).

Gene Ontology ID	Gene Ontology annotation	Odds ratio	Adjusted *p* value	Number of enriched genes/Total number of genes in annotation set (*n*)
**Enriched terms consisting of genes that were up-regulated (n = 570)**				
GO:0046339	diacylglycerol metabolic process	41.13	0.044	4/7
GO:0050727	regulation of inflammatory response	4.21	0.044	14/125
GO:0052548	regulation of endopeptidase activity	3.70	0.003	21/210
GO:0052547	regulation of peptidase activity	3.61	0.003	21/215
GO:0006954	inflammatory response	3.18	0.012	23/264
GO:0031347	regulation of defense response	2.86	0.007	28/354
GO:0009611	response to wounding	2.78	<0.001	56/744
GO:0050878	regulation of body fluid levels	2.66	0.002	36/490
GO:0007596	blood coagulation	2.64	0.005	33/451
GO:0050817	coagulation	2.62	0.005	33/453
GO:0007599	hemostasis	2.61	0.007	33/455
GO:0001775	cell activation	2.61	0.002	37/512
GO:0080134	regulation of response to stress	2.46	0.001	43/631
GO:0016192	vesicle-mediated transport	2.33	0.005	42/646
**Enriched terms consisting of genes that were down-regulated (n = 763)**				
GO:0006405	RNA export from nucleus	5.78	0.011	13/68
GO:0015931	nucleobase-containing compound transport	5.08	0.004	16/93
GO:0050657	nucleic acid transport	5.05	0.028	13/76
GO:0050658	RNA transport	5.05	0.028	13/76
GO:0051236	establishment of RNA localization	5.05	0.028	13/76
GO:0006403	RNA localization	4.75	0.05	13/80
GO:0000956	nuclear-transcribed mRNA catabolic process	4.40	0.005	18/118
GO:0006402	mRNA catabolic process	4.00	0.012	18/128
GO:0006401	RNA catabolic process	3.67	0.011	20/153
GO:0006396	RNA processing	2.81	<0.001	43/419
GO:0006412	translation	2.63	0.048	28/287
GO:0016071	mRNA metabolic process	2.60	0.002	40/417
GO:0022402	cell cycle process	2.13	0.001	65/819
GO:0005634	nucleus	2.08	<0.001	244/3551
GO:0000278	mitotic cell cycle	2.07	0.028	49/628
GO:0007049	cell cycle	2.05	0.001	76/999
GO:0044428	nuclear part	2.01	<0.001	130/1798

### Validation of microarray using qRT-PCR analysis

Twenty eight differentially expressed genes (±0.6 Log_2_ fold change and ≥4 microarray intensity expression) were chosen for microarray data validation using qRT-PCR. A total of ninety six samples (a subset of the 154 women) were selected for qRT-PCR validation: 41 samples from women who had sPTB within 48 hours were used and 55 samples were randomly chosen from the 106 women who did not deliver within 48 hours. After adjusting for gestational age at presentation, qRT-PCR confirmed the differential expression of 20 genes (*p*<0.05) and the direction of change in all 28 genes were 100% consistent with the microarray analysis (Table S3 in File S1). There was a significant correlation between microarray and qRT-PCR data (Pearson's *r* = 0.956, *p*<0.001).

### Generation of models to predict sPTB within 48 hours

**Figure 4:**
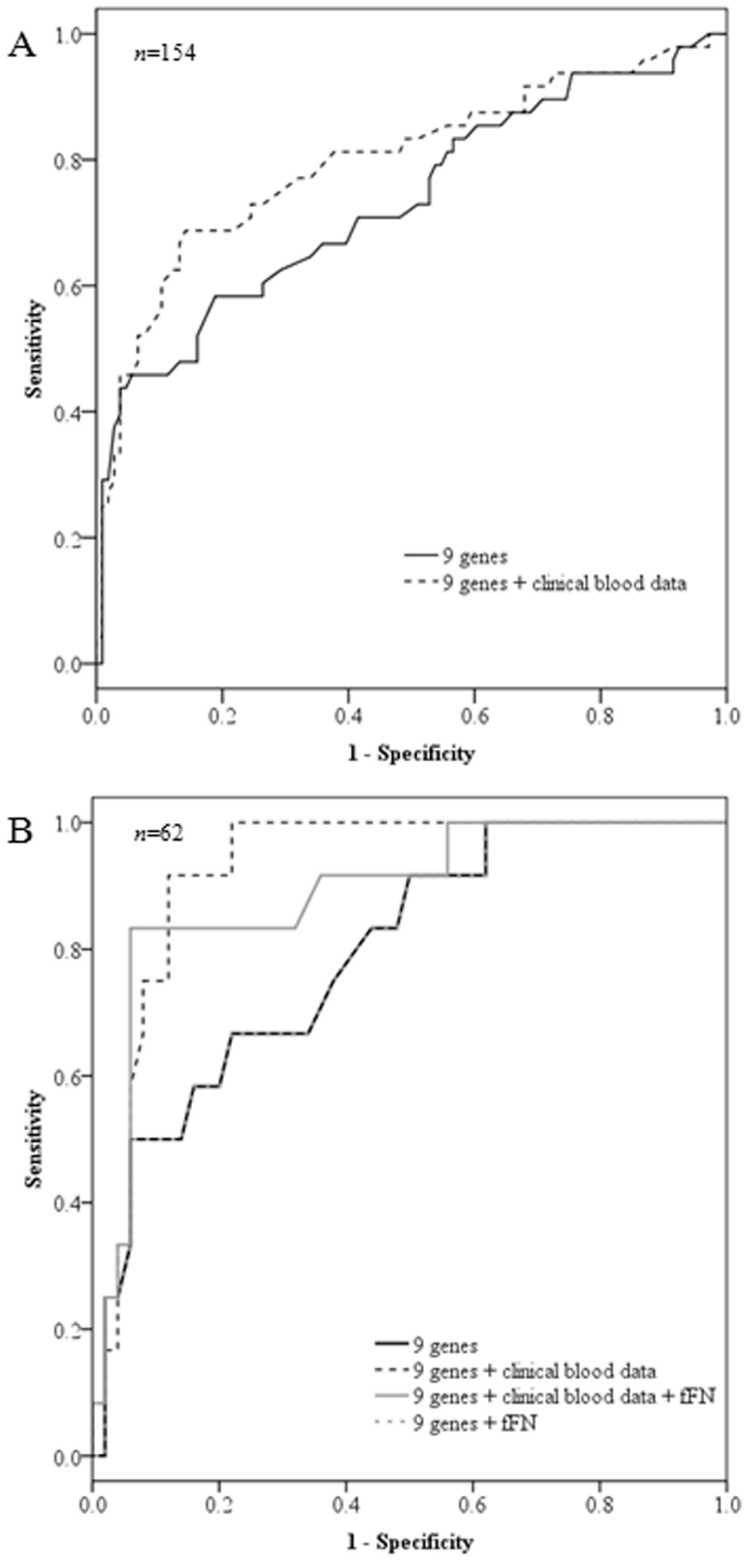
Receiver operator characteristics curves displaying the performances of the random forest classifier models. (**A**) Models using the top nine genes, with and without peripheral clinical blood data in 154 women. (**B**) Models developed using the top nine genes, fetal fibronectin (fFN) and clinical blood data in 62 women. The lines for nine genes only and nine genes with fFN are superimposed.

**Figure 5:**
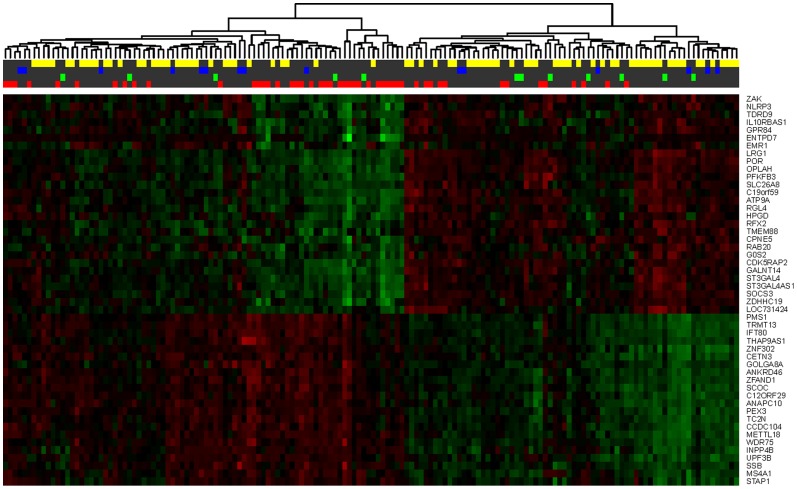
This heat map of the top 50 differentially expressed genes (highest fold changes) displays the hierarchical clustering, consistency and regulation of the gene expression levels. Elevated gene expressions are indicated in green; down-regulated gene expressions are indicated in red. The colors on the top bars represent women who had a spontaneous preterm birth within 48 hours (red), preterm delivery between 2 to 7 days (green), preterm delivery between 7 days and within 37 weeks of gestation (blue) and women who delivered at term (yellow).

Optimal random forest classifier model performances was achieved using the top nine differentially expressed genes (*ZDHHC19, HPGD, GPR84, OPLAH, METTL18, TDRD9, ATP9A, GALNT14* and *GOLGA8A*; ranked by decreasing magnitude of fold change, *Limma* FDR <0.05) with 100 decision trees. We also evaluated whether the inclusion of the five peripheral clinical blood data (total leukocyte, neutrophil, lymphocyte and monocyte counts and hemoglobin levels) in the model improved prediction. Table 5 displays the random forest classifier performances generated with 154 samples using the top nine genes with and without clinical blood data (Figure 4A). A representative heat map of the top 50 genes demonstrating the highest fold changes (*Limma* FDR <0.05) is shown in Figure 5.

**Table 5:** Predicting spontaneous preterm birth within 48(with and without peripheral clinical blood data) and comparing the predictive efficacies of fetal fibronectin and the random forest classifier models (top 9 genes, with or without peripheral clinical blood data and fFN) to predict spontaneous preterm birth within 48 hours in 62 women.

	Top 9 Genes		Fetal Fibronectin only	Top 9 Genes			
**Women, ** ***n***	154	154	62	62	62	62	62
**Clinical Blood Data (included in model)**	Yes	No	-	Yes	No	Yes	No
**Fetal Fibronectin (included in model)**	-	-	-	-	-	Yes	Yes
**ROC-AUC**	0.798	0.733	-	0.926	0.794	0.888	0.794
***p*** **-value**	<0.001	<0.001	-	0.002	<0.001	<0.001	0.002
**Classifier Score Cut Off**	0.400	0.530	-	0.450	0.540	0.500	0.590
**Sensitivity (%)**	70.8	54.2	83.3	91.7	58.3	83.3	58.3
**Specificity (%)**	75.5	83.0	66.0	88.0	80.0	94.0	84.0
**Positive Predictive Value (%)**	56.7	59.1	37.0	64.7	41.2	76.9	46.7
**Negative Predictive Value (%)**	85.1	80.0	94.3	97.8	88.9	95.9	89.4
**False Positive (%)**	24.5	17.0	34.0	12.0	20.0	6.0	16.0
**False Negative (%)**	29.2	45.8	16.7	8.3	41.7	16.7	41.7

### Comparison of gene signatures and fFN

To obtain an unbiased performance comparison between the microarray genomic signature and fFN, separate classifier models were constructed on the subset of patients who had corresponding fFN test results (n = 62). Table 5 also displays the comparison of the predictive efficacies of the random forest classifier models (using top nine genes, with and without clinical blood data, and with and without fFN test results included in the model) and the stand-alone fFN test to predict sPTB within 48 hours in 62 women (Figure 4B).

## Discussion

This study has utilized microarrays to provide novel genomic insights of sPTB and our *Limma* analysis revealed 469 differentially expressed genes associated with sPTB within 48 hours in women with TPTL. PTB is a heterogeneous condition with varying etiologies. We accounted for known causes of PTB with our exclusion criteria and attempted to discover a blood-based gene signature to predict imminent idiopathic sPTB. Labor is an inflammatory process with elevated levels of maternal circulating leukocytes [41], [42] and increased leukocyte infiltration into the myometrium, decidua and cervix before and during labor [43]–[45]. Thus, it was unsurprising to observe elevated whole blood mRNA expression of cytokines (*IL-1beta, OSM*), cytokine/chemokine receptors (*IL-4R, IL10RB, TNFRSF10D, LTBR, CCR1, CXCR1*), genes involved in cytokine signaling pathways (*JAK3, TNIP2, SOCS3*) and related transcriptional factors (*NFKB1, JUNB*) in our *Limma* differential analysis as well as inflammation-related clusters (Reactome) and enriched GO terms (FuncAssociate) in women who had sPTB within 48 hours. This suggests that a repertoire of inflammatory markers in the blood are differentially expressed 48 hours prior to labor and associated with impending delivery.

There is a wealth of information linking infection and PTB (reviewed in [4], [46], [47]). Despite our attempt to study idiopathic sPTB, these participants may have undiagnosed sub-clinical infection. Thus, the differential expression of infection- or inflammation-related genes in our study could be attributed to a combination of infection and labor. For example, inflammatory-related genes and their respective proteins such as *NFKB1* [48], *CCR1* [49], *SOCS3, JAK3* [50], *JUNB* [51], *RHOG* [52], *TIMP1* [53], *IL1beta* [54]–[57], ITGAM [58], CD44 [59], [60], TLR5 [61]–[63] and CXCR1 [64], [65] from our Reactome analysis are known to be up-regulated in reproductive tissues and other biologic fluids during term labor or PTL with or without infection. CD63 was increased in our study but fetal blood CD63 was not associated with sPTB [66]. Whole blood is widely studied [67], [68] because it serves as reservoir that collects information from various physiologic processes such as immune response and cell-cell communication. It can be assumed that differentially expressed whole blood mRNAs are in part, contributed by activated maternal peripheral leukocytes with impending labor [69], [70]. We observed an increase in peripheral blood leukocyte counts in women who delivered within 48 hours and this increase appears to be mainly contributed by neutrophils. This phenomenon agrees with the study by Campbell et al where women with TPTL without infection had increased leukocyte counts [71] and supports previous studies which reported increased circulating maternal leukocytes before and during labor [41], [42], [72]–[75]. Thus, our study has identified novel genes, most likely originating from leukocytes, which may serve as therapeutic targets for sPTB prevention.

We decided to focus our discussion on the top three differentially expressed genes, *ZDHHC19, HPGD* and *GPR84*. DHHC19 (*ZDHHC19*; 2.3-fold increase) belongs to a family of palmitoyltransferases/acyltransferases that catalyzes post-translational attachment of palmitic acid groups onto cysteine residues via thioester linkage (i.e. palmitoylation) on newly synthesized protein substrates, thereby enhancing the association of these palmitoylated proteins with lipid bilayers of the plasma membrane and transport vesicles [76], [77]. Protein palmitoylation is essential for a diverse range of cellular processes such as protein-protein interactions, protein trafficking, stability, degradation and intra-cellular localization [76], [78]. Palmitoylation by DHHC19 increased R-Ras association with plasma membranes and lipid rafts in COS7 cells [77] as well as enhanced phosphodiesterase 10A2 (PDE10A2) binding to plasma membranes in HEK293 cells and mouse primary striatal neurons [79]. The up-regulation of *ZDHHC19* in women who delivered within 48 hours could be linked to protein palmitoylation and the regulation of signaling pathways involving R-Ras and PDE10A2 in peripheral leukocytes.

HPGD demonstrated a 2.1-fold increase in women who had a sPTB within 48 hours. HPGD catabolizes prostaglandin into inactive keto-metabolites. HPGD is expressed in reproductive tissues, especially in the myometrium [80], chorion [81] and cervix [82]. HPGD is significantly decreased during term and PTL [80]–[83], thus promoting the effect of prostaglandin during labor [84], [85]. The increase in whole blood HPGD mRNA may be a homeostatic response to the elevated concentration of prostaglandin associated with impending labor onset [86], [87].

GPR84 (2.0-fold increase) is a G protein-coupled receptor highly expressed in immune cells such as microglia, monocytes and neutrophils [88]–[91]. It is activated by medium-chain (C_9_-C_14_) free fatty acids (FFAs) such as capric, undecanoic and lauric acids [89] but not medium-chain triglycerides [92]. Hyper production of IL-4 occurs in GPR84^−/−^ activated T-cells displaying T helper 2 phenotype [90]. Lipopolysaccharide (LPS) increases GPR84 expression in monocytes and microglia [88], [89]. In addition, medium-chain FFAs can enhance LPS-stimulated secretion of the pro-inflammatory cytokine, IL-12 p40, via GPR84 in monocytes [89] while TNF and IL-1 can also increase GPR84 mRNA expression in microglia [88]. The increase in GPR84 mRNA in our study could be contributed by leukocytes which are stimulated by IL-1 and TNF in the serum [93], [94], or suggests the potential novel involvement of medium-chain FFAs in signaling pathways and immunologic regulation prior to and during labor.

From our bioinformatics modeling of all 154 women, a set of nine genes coupled with clinical blood data could classify women who would or would not have a sPTB within 48 hours of hospital admission with 70.8% sensitivity and 75.5% specificity. The next step was to benchmark the performance of the stand-alone fFN test and our nine gene signature. fFN was only performed on 62 eligible women. When we modeled data from these 62 women, the nine genes coupled with clinical blood data was the best performing model with the highest area under ROC curve. It had improved sensitivity (91.7% vs 83.3%) and specificity (88.0% vs 66.0%) compared with the stand-alone fFN test. The effect of fFN was generally minimal (identical or lower area under the ROC curves) when fFN was included as a feature in the classifier models. The minimal contribution of fFN in the models may be because whole blood mRNA and fFN represent two fundamentally different elements of the parturition process - fFN indicates a disruption of the maternal-fetal interface and activated leukocytes contribute to the process of labor. However, it was also interesting to note that the addition of fFN into the model shifted the performance of the original model (nine genes and clinical blood data) from being a highly sensitive test to a more specific test (nine genes, clinical blood data and fFN) to predict sPTB. This influence may be attributed to the high specificity (NPV) of the fFN test [15].

The heterogeneity of sPTB and the inability to identify sub clinical patients may explain the small fold changes observed in this study and why no significant pathway analysis was obtained. This is a common challenge in PTL discovery or diagnostic studies. Moreover, our findings are limited to our institution with a homogenous population and therefore, the generalizability of our gene signatures is limited. Nevertheless, this gene expression study provides a broad overview of the mechanisms of sPTB and generates future hypotheses to investigate molecular interactions of various cell types (e.g. leukocytes) and reproductive tissues (e.g. placenta and myometrium) associated with sPTB. The predictive efficacy of our nine gene signature coupled with clinical blood data outperformed the fFN test and highlights the advantage of utilizing a blood-based diagnostic test for sPTB where all women could be tested. This study may lead the way for a blood-based systems biology approach to sPTB in the future.

## Supporting Information

File S1 Contains the following files: **Table S1**. Clinical data of 154 women (GEO microarray submission). **Table S2**. The list of 469 significant differentially expressed genes obtained using *Limma* ranked by magnitude of fold change. **Table S3**. Microarray and quantitative real time-PCR of the selected 28 genes.
(DOCX)
