# Supplementary material for: Whole Blood Gene Expression Profile Associated with Spontaneous Preterm Birth in Women with Threatened Preterm Labor
Source: PLoS One. 2014 May 14;9(5):e96901. doi: 10.1371/journal.pone.0096901 (PMC4020779; doi:10.1371/journal.pone.0096901)
Supplement: File S1 — Contains the following files: Table S1. Clinical data of 154 women (GEO microarray submission). Table S2. The list of 469 significant differentially expressed genes obtained using Limma ranked by magnitude of fold change. Table S3. Microarray and quantitative real time-PCR of the selected 28 genes. (DOCX) [file pone.0096901.s001.docx]

**Table S1. Clinical data of 154 women (GEO microarray submission)**

| **Sample** | **Title** | **CEL file** | **Gestational Age at Sampling** | **Gestational Age at Delivery** | **Del <48hrs** | **Del <7days** | **PTB <37 weeks** |
| --- | --- | --- | --- | --- | --- | --- | --- |
| T-102 | wholeblood_woman_T-102 | U133Plus2_011207W_SL54_T102 | 31.14 | 31.14 | 1 | 1 | 1 |
| T-129 | wholeblood_woman_T-129 | U133Plus2_011907W_SL70_T129 | 32.43 | 37.71 | 0 | 0 | 0 |
| T-150 | wholeblood_woman_T-150 | U133Plus2_101707W(2)_SL35_T150 | 24.86 | 24.86 | 1 | 1 | 1 |
| T-072 | wholeblood_woman_T-072 | U133Plus2_101707W(2)_SL48_T072 | 32.29 | 34.29 | 0 | 0 | 1 |
| T-046 | wholeblood_woman_T-046 | U133Plus2_010307W_SL28_T046 | 31.29 | 37.43 | 0 | 0 | 0 |
| T-243 | wholeblood_woman_T-243 | U133Plus2_101007W_SL20_T243 | 26.00 | 37.43 | 0 | 0 | 0 |
| T-194 | wholeblood_woman_T-194 | U133Plus2_102507W(2)_SL56_T194 | 33.86 | 38.00 | 0 | 0 | 0 |
| T-188 | wholeblood_woman_T-188 | U133Plus2_102507W(2)_SL53_T188 | 34.29 | 37.57 | 0 | 0 | 0 |
| T-109 | wholeblood_woman_T-109 | U133Plus2_011907W_SL61_T109 | 32.00 | 39.00 | 0 | 0 | 0 |
| T-160 | wholeblood_woman_T-160 | U133Plus2_101707W(2)_SL37_T160 | 26.57 | 27.14 | 0 | 1 | 1 |
| T-268 | wholeblood_woman_T-268 | U133Plus2_103107W(2)_SL74_T268 | 26.14 | 26.14 | 1 | 1 | 1 |
| T-048 | wholeblood_woman_T-048 | U133Plus2_010307W_SL29_T048 | 34.43 | 34.71 | 1 | 1 | 1 |
| T-153 | wholeblood_woman_T-153 | U133Plus2_101707W(2)_SL36_T153 | 29.14 | 31.86 | 0 | 0 | 1 |
| T-085 | wholeblood_woman_T-085 | U133Plus2_011207W_SL48_T085 | 33.43 | 33.57 | 1 | 1 | 1 |
| T-090 | wholeblood_woman_T-090 | U133Plus2_011207W_SL51_T090 | 30.43 | 36.86 | 0 | 0 | 1 |
| T-238 | wholeblood_woman_T-238 | U133Plus2_101007W_SL17_T238 | 31.14 | 38.00 | 0 | 0 | 0 |
| T-007 | wholeblood_woman_T-007 | U133Plus2_101707W_SL25_T007 | 32.29 | 36.43 | 0 | 0 | 1 |
| T-045 | wholeblood_woman_T-045 | U133Plus2_010307W_SL27_T045 | 30.14 | 37.57 | 0 | 0 | 0 |
| T-157 | wholeblood_woman_T-157 | U133Plus2_011907W_SL82_T157 | 27.57 | 40.71 | 0 | 0 | 0 |
| T-023 | wholeblood_woman_T-023 | U133Plus2_112505_XW_SL06B | 31.86 | 31.86 | 1 | 1 | 1 |
| T-053 | wholeblood_woman_T-053 | U133Plus2_011207W_SL33_T053 | 28.71 | 39.71 | 0 | 0 | 0 |
| T-004 | wholeblood_woman_T-004 | U133Plus2_120606W_SL03_T004 | 32.14 | 37.43 | 0 | 0 | 0 |
| T-273 | wholeblood_woman_T-273 | U133Plus2_103107W(2)_SL77_T273 | 25.71 | 39.43 | 0 | 0 | 0 |
| T-204 | wholeblood_woman_T-204 | U133Plus2_102507W(2)_SL61_T204 | 32.86 | 33.14 | 1 | 1 | 1 |
| T-141 | wholeblood_woman_T-141 | U133Plus2_101707W(2)_SL33_T141 | 26.86 | 40.00 | 0 | 0 | 0 |
| T-166 | wholeblood_woman_T-166 | U133Plus2_101707W(2)_SL41_T166 | 32.71 | 38.00 | 0 | 0 | 0 |
| T-028 | wholeblood_woman_T-028 | U133Plus2_120606W_SL16_T028 | 29.57 | 29.86 | 1 | 1 | 1 |
| T-006 | wholeblood_woman_T-006 | U133Plus2_120606W_SL05_T006 | 34.71 | 34.86 | 1 | 1 | 1 |
| T-154 | wholeblood_woman_T-154 | U133Plus2_011907W_SL80_T154 | 28.00 | 28.43 | 0 | 1 | 1 |
| T-255 | wholeblood_woman_T-255 | U133Plus2_103107W(2)_SL69_T255 | 29.43 | 39.57 | 0 | 0 | 0 |
| T-158 | wholeblood_woman_T-158 | U133Plus2_103107W(2)_SL92_T158 | 32.57 | 32.57 | 1 | 1 | 1 |
| T-168 | wholeblood_woman_T-168 | U133Plus2_103107W(2)_SL93_T168 | 33.71 | 33.86 | 1 | 1 | 1 |
| T-016 | wholeblood_woman_T-016 | U133Plus2_112505_XW_SL04B | 34.57 | 40.14 | 0 | 0 | 0 |
| T-249 | wholeblood_woman_T-249 | U133Plus2_101007W_SL22_T249 | 35.43 | 35.71 | 1 | 1 | 1 |
| T-193 | wholeblood_woman_T-193 | U133Plus2_102507W(2)_SL55_T193 | 29.14 | 29.14 | 1 | 1 | 1 |
| T-136 | wholeblood_woman_T-136 | U133Plus2_101707W(2)_SL32_T136 | 31.71 | 38.57 | 0 | 0 | 0 |
| T-290 | wholeblood_woman_T-290 | U133Plus2_103107W(2)_SL87_T290 | 30.14 | 30.43 | 1 | 1 | 1 |
| T-225 | wholeblood_woman_T-225 | U133Plus2_101007W_SL08_T225 | 24.43 | 24.43 | 1 | 1 | 1 |
| T-127 | wholeblood_woman_T-127 | U133Plus2_011907W_SL69_T127 | 27.14 | 28.00 | 0 | 1 | 1 |
| T-272 | wholeblood_woman_T-272 | U133Plus2_103107W(2)_SL76_T272 | 32.43 | 36.71 | 0 | 0 | 1 |
| T-097 | wholeblood_woman_T-097 | U133Plus2_011207W_SL53_T097 | 27.71 | 41.00 | 0 | 0 | 0 |
| T-142 | wholeblood_woman_T-142 | U133Plus2_011907W_SL76_T142 | 33.86 | 33.86 | 1 | 1 | 1 |
| T-070 | wholeblood_woman_T-070 | U133Plus2_011207W_SL43_T070 | 30.14 | 38.71 | 0 | 0 | 0 |
| T-221 | wholeblood_woman_T-221 | U133Plus2_101007W_SL06_T221 | 31.86 | 31.86 | 1 | 1 | 1 |
| T-002 | wholeblood_woman_T-002 | U133Plus2_120606W_SL02_T002 | 28.57 | 40.00 | 0 | 0 | 0 |
| T-079 | wholeblood_woman_T-079 | U133Plus2_011207W_SL46_T079 | 30.14 | 39.43 | 0 | 0 | 0 |
| T-234 | wholeblood_woman_T-234 | U133Plus2_101007W_SL14_T234 | 33.00 | 33.57 | 0 | 1 | 1 |
| T-262 | wholeblood_woman_T-262 | U133Plus2_103107W(2)_SL72_T262 | 34.71 | 39.00 | 0 | 0 | 0 |
| T-035 | wholeblood_woman_T-035 | U133Plus2_120606W_SL20_T035 | 32.57 | 39.71 | 0 | 0 | 0 |
| T-022 | wholeblood_woman_T-022 | U133Plus2_101707W_SL26_T022 | 31.00 | 36.43 | 0 | 0 | 1 |
| T-263 | wholeblood_woman_T-263 | U133Plus2_103107W(2)_SL73_T263 | 33.29 | 39.29 | 0 | 0 | 0 |
| T-042 | wholeblood_woman_T-042 | U133Plus2_010307W_SL25_T042 | 33.86 | 37.00 | 0 | 0 | 0 |
| T-288 | wholeblood_woman_T-288 | U133Plus2_111307W(2)_SL95_T288 | 33.57 | 39.57 | 0 | 0 | 0 |
| T-169 | wholeblood_woman_T-169 | U133Plus2_103107W(2)_SL94_T169 | 32.57 | 32.57 | 1 | 1 | 1 |
| T-068 | wholeblood_woman_T-068 | U133Plus2_011207W_SL41_T068 | 34.43 | 34.43 | 1 | 1 | 1 |
| T-055 | wholeblood_woman_T-055 | U133Plus2_011207W_SL35_T055 | 33.14 | 37.57 | 0 | 0 | 0 |
| T-197 | wholeblood_woman_T-197 | U133Plus2_102507W(2)_SL58_T197 | 29.86 | 31.14 | 0 | 0 | 1 |
| T-049 | wholeblood_woman_T-049 | U133Plus2_010307W_SL30_T049 | 35.14 | 39.00 | 0 | 0 | 0 |
| T-212 | wholeblood_woman_T-212 | U133Plus2_101007W_SL01_T212 | 32.57 | 32.71 | 1 | 1 | 1 |
| T-237 | wholeblood_woman_T-237 | U133Plus2_101007W_SL16_T237 | 33.29 | 34.00 | 0 | 1 | 1 |
| T-119 | wholeblood_woman_T-119 | U133Plus2_011907W_SL65_T119 | 32.43 | 40.29 | 0 | 0 | 0 |
| T-274 | wholeblood_woman_T-274 | U133Plus2_103107W(2)_SL78_T274 | 26.14 | 26.14 | 1 | 1 | 1 |
| T-096 | wholeblood_woman_T-096 | U133Plus2_101707W(2)_SL31_T096 | 33.14 | 37.71 | 0 | 0 | 0 |
| T-300 | wholeblood_woman_T-300 | U133Plus2_103107W(2)_SL90_T300 | 25.29 | 25.29 | 1 | 1 | 1 |
| T-175 | wholeblood_woman_T-175 | U133Plus2_101707W(2)_SL45_T175 | 33.29 | 35.14 | 0 | 0 | 1 |
| T-013 | wholeblood_woman_T-013 | U133Plus2_112505_XW_SL03B | 32.57 | 32.57 | 1 | 1 | 1 |
| T-224 | wholeblood_woman_T-224 | U133Plus2_101007W_SL07_T224 | 31.00 | 31.14 | 1 | 1 | 1 |
| T-014 | wholeblood_woman_T-014 | U133Plus2_120606W_SL10_T014 | 32.00 | 40.29 | 0 | 0 | 0 |
| T-155 | wholeblood_woman_T-155 | U133Plus2_011907W_SL81_T155 | 29.00 | 38.57 | 0 | 0 | 0 |
| T-217 | wholeblood_woman_T-217 | U133Plus2_101007W_SL03_T217 | 31.86 | 36.14 | 0 | 0 | 1 |
| T-183 | wholeblood_woman_T-183 | U133Plus2_102507W(2)_SL52_T183 | 34.57 | 34.57 | 1 | 1 | 1 |
| T-284 | wholeblood_woman_T-284 | U133Plus2_103107W(2)_SL84_T284 | 28.43 | 39.00 | 0 | 0 | 0 |
| T-172 | wholeblood_woman_T-172 | U133Plus2_111307W(2)_SL96_T172 | 31.29 | 40.00 | 0 | 0 | 0 |
| T-173 | wholeblood_woman_T-173 | U133Plus2_101707W(2)_SL43_T173 | 28.71 | 38.43 | 0 | 0 | 0 |
| T-275 | wholeblood_woman_T-275 | U133Plus2_103107W(2)_SL79_T275 | 26.43 | 26.86 | 0 | 1 | 1 |
| T-144 | wholeblood_woman_T-144 | U133Plus2_011907W_SL77_T144 | 26.29 | 26.71 | 0 | 1 | 1 |
| T-278 | wholeblood_woman_T-278 | U133Plus2_103107W(2)_SL80_T278 | 31.43 | 40.00 | 0 | 0 | 0 |
| T-170 | wholeblood_woman_T-170 | U133Plus2_101707W(2)_SL42_T170 | 29.00 | 39.86 | 0 | 0 | 0 |
| T-089 | wholeblood_woman_T-089 | U133Plus2_011207W_SL50_T089 | 31.43 | 38.00 | 0 | 0 | 0 |
| T-203 | wholeblood_woman_T-203 | U133Plus2_102507W(2)_SL60_T203 | 25.86 | 39.00 | 0 | 0 | 0 |
| T-051 | wholeblood_woman_T-051 | U133Plus2_011207W_SL32_T051 | 32.00 | 33.00 | 0 | 1 | 1 |
| T-003 | wholeblood_woman_T-003 | U133Plus2_112505_XW_SL01B | 28.57 | 38.57 | 0 | 0 | 0 |
| T-012 | wholeblood_woman_T-012 | U133Plus2_120606W_SL09_T012 | 32.57 | 37.57 | 0 | 0 | 0 |
| T-252 | wholeblood_woman_T-252 | U133Plus2_101007W_SL24_T252 | 28.71 | 29.14 | 0 | 1 | 1 |
| T-106 | wholeblood_woman_T-106 | U133Plus2_011907W_SL59_T106 | 32.86 | 33.00 | 1 | 1 | 1 |
| T-077 | wholeblood_woman_T-077 | U133Plus2_101707W(2)_SL49_T077 | 32.14 | 33.14 | 0 | 1 | 1 |
| T-270 | wholeblood_woman_T-270 | U133Plus2_103107W(2)_SL75_T270 | 35.57 | 38.86 | 0 | 0 | 0 |
| T-064 | wholeblood_woman_T-064 | U133Plus2_011207W_SL38_T064 | 34.57 | 38.00 | 0 | 0 | 0 |
| T-123 | wholeblood_woman_T-123 | U133Plus2_011907W_SL67_T123 | 32.14 | 32.14 | 1 | 1 | 1 |
| T-075 | wholeblood_woman_T-075 | U133Plus2_011207W_SL44_T075 | 32.29 | 39.00 | 0 | 0 | 0 |
| T-124 | wholeblood_woman_T-124 | U133Plus2_011907W_SL68_T124 | 34.43 | 34.57 | 1 | 1 | 1 |
| T-020 | wholeblood_woman_T-020 | U133Plus2_120606W_SL14_T020 | 27.29 | 28.14 | 0 | 1 | 1 |
| T-044 | wholeblood_woman_T-044 | U133Plus2_010307W_SL26_T044 | 26.57 | 26.86 | 1 | 1 | 1 |
| T-218 | wholeblood_woman_T-218 | U133Plus2_101007W_SL04_T218 | 24.86 | 25.29 | 0 | 1 | 1 |
| T-287 | wholeblood_woman_T-287 | U133Plus2_103107W(2)_SL86_T287 | 35.00 | 37.86 | 0 | 0 | 0 |
| T-140 | wholeblood_woman_T-140 | U133Plus2_011907W_SL75_T140 | 33.00 | 40.29 | 0 | 0 | 0 |
| T-030 | wholeblood_woman_T-030 | U133Plus2_120606W_SL18_T030 | 34.71 | 38.14 | 0 | 0 | 0 |
| T-180 | wholeblood_woman_T-180 | U133Plus2_101707W(2)_SL47_T180 | 35.57 | 35.71 | 1 | 1 | 1 |
| T-248 | wholeblood_woman_T-248 | U133Plus2_101007W_SL21_T248 | 31.86 | 31.86 | 1 | 1 | 1 |
| T-067 | wholeblood_woman_T-067 | U133Plus2_011207W_SL40_T067 | 32.00 | 42.14 | 0 | 0 | 0 |
| T-041 | wholeblood_woman_T-041 | U133Plus2_120606W_SL24_T041 | 31.00 | 38.00 | 0 | 0 | 0 |
| T-018 | wholeblood_woman_T-018 | U133Plus2_120606W_SL13_T018 | 27.86 | 27.86 | 1 | 1 | 1 |
| T-054 | wholeblood_woman_T-054 | U133Plus2_011207W_SL34_T054 | 33.71 | 37.86 | 0 | 0 | 0 |
| T-021 | wholeblood_woman_T-021 | U133Plus2_112505_XW_SL05B | 35.00 | 37.00 | 0 | 0 | 0 |
| T-148 | wholeblood_woman_T-148 | U133Plus2_101707W(2)_SL34_T148 | 33.29 | 33.29 | 1 | 1 | 1 |
| T-017 | wholeblood_woman_T-017 | U133Plus2_120606W_SL12_T017 | 26.29 | 39.86 | 0 | 0 | 0 |
| T-286 | wholeblood_woman_T-286 | U133Plus2_103107W(2)_SL85_T286 | 30.29 | 32.71 | 0 | 0 | 1 |
| T-147 | wholeblood_woman_T-147 | U133Plus2_011907W_SL79_T147 | 32.14 | 32.43 | 1 | 1 | 1 |
| T-078 | wholeblood_woman_T-078 | U133Plus2_011207W_SL45_T078 | 30.71 | 38.71 | 0 | 0 | 0 |
| T-069 | wholeblood_woman_T-069 | U133Plus2_011207W_SL42_T069 | 28.00 | 37.57 | 0 | 0 | 0 |
| T-001 | wholeblood_woman_T-001 | U133Plus2_120606W_SL01_T001 | 32.00 | 32.00 | 1 | 1 | 1 |
| T-103 | wholeblood_woman_T-103 | U133Plus2_011907W_SL58_T103 | 34.43 | 34.43 | 1 | 1 | 1 |
| T-165 | wholeblood_woman_T-165 | U133Plus2_101707W(2)_SL40_T165 | 30.00 | 39.86 | 0 | 0 | 0 |
| T-104 | wholeblood_woman_T-104 | U133Plus2_011207W_SL55_T104 | 33.57 | 40.14 | 0 | 0 | 0 |
| T-031 | wholeblood_woman_T-031 | U133Plus2_120606W_SL19_T031 | 34.14 | 34.29 | 1 | 1 | 1 |
| T-029 | wholeblood_woman_T-029 | U133Plus2_120606W_SL17_T029 | 34.43 | 38.14 | 0 | 0 | 0 |
| T-133 | wholeblood_woman_T-133 | U133Plus2_011907W_SL72_T133 | 33.00 | 37.00 | 0 | 0 | 0 |
| T-120 | wholeblood_woman_T-120 | U133Plus2_011907W_SL66_T120 | 25.29 | 40.71 | 0 | 0 | 0 |
| T-161 | wholeblood_woman_T-161 | U133Plus2_101707W(2)_SL38_T161 | 29.86 | 41.00 | 0 | 0 | 0 |
| T-071 | wholeblood_woman_T-071 | U133Plus2_101707W_SL28_T071 | 23.71 | 39.14 | 0 | 0 | 0 |
| T-279 | wholeblood_woman_T-279 | U133Plus2_103107W(2)_SL81_T279 | 35.00 | 35.29 | 1 | 1 | 1 |
| T-231 | wholeblood_woman_T-231 | U133Plus2_101007W_SL11_T231 | 33.71 | 36.86 | 0 | 0 | 1 |
| T-025 | wholeblood_woman_T-025 | U133Plus2_120606W_SL15_T025 | 33.29 | 40.00 | 0 | 0 | 0 |
| T-251 | wholeblood_woman_T-251 | U133Plus2_101007W_SL23_T251 | 31.14 | 40.43 | 0 | 0 | 0 |
| T-066 | wholeblood_woman_T-066 | U133Plus2_011207W_SL39_T066 | 34.00 | 39.14 | 0 | 0 | 0 |
| T-232 | wholeblood_woman_T-232 | U133Plus2_101007W_SL12_T232 | 35.71 | 35.71 | 1 | 1 | 1 |
| T-107 | wholeblood_woman_T-107 | U133Plus2_011907W_SL60_T107 | 31.71 | 41.00 | 0 | 0 | 0 |
| T-111 | wholeblood_woman_T-111 | U133Plus2_101707W_SL29_T111 | 34.00 | 34.14 | 1 | 1 | 1 |
| T-094 | wholeblood_woman_T-094 | U133Plus2_011207W_SL52_T094 | 28.71 | 39.29 | 0 | 0 | 0 |
| T-005 | wholeblood_woman_T-005 | U133Plus2_120606W_SL04_T005 | 33.14 | 38.43 | 0 | 0 | 0 |
| T-132 | wholeblood_woman_T-132 | U133Plus2_011907W_SL71_T132 | 34.71 | 38.57 | 0 | 0 | 0 |
| T-134 | wholeblood_woman_T-134 | U133Plus2_011907W_SL73_T134 | 32.71 | 41.57 | 0 | 0 | 0 |
| T-113 | wholeblood_woman_T-113 | U133Plus2_011907W_SL63_T113 | 34.00 | 40.43 | 0 | 0 | 0 |
| T-058 | wholeblood_woman_T-058 | U133Plus2_011207W_SL37_T058 | 30.29 | 37.86 | 0 | 0 | 0 |
| T-011 | wholeblood_woman_T-011 | U133Plus2_120606W_SL08_T011 | 31.57 | 37.00 | 0 | 0 | 0 |
| T-116 | wholeblood_woman_T-116 | U133Plus2_011907W_SL64_T116 | 25.43 | 25.43 | 1 | 1 | 1 |
| T-008 | wholeblood_woman_T-008 | U133Plus2_120606W_SL06_T008 | 25.71 | 39.29 | 0 | 0 | 0 |
| T-162 | wholeblood_woman_T-162 | U133Plus2_101707W(2)_SL39_T162 | 30.71 | 39.00 | 0 | 0 | 0 |
| T-226 | wholeblood_woman_T-226 | U133Plus2_101007W_SL09_T226 | 30.14 | 30.29 | 1 | 1 | 1 |
| T-195 | wholeblood_woman_T-195 | U133Plus2_102507W(2)_SL57_T195 | 27.14 | 27.14 | 1 | 1 | 1 |
| T-009 | wholeblood_woman_T-009 | U133Plus2_120606W_SL07_T009 | 27.00 | 38.00 | 0 | 0 | 0 |
| T-039 | wholeblood_woman_T-039 | U133Plus2_120606W_SL22_T039 | 32.57 | 41.57 | 0 | 0 | 0 |
| T-015 | wholeblood_woman_T-015 | U133Plus2_120606W_SL11_T015 | 29.86 | 31.57 | 0 | 0 | 1 |
| T-112 | wholeblood_woman_T-112 | U133Plus2_011907W_SL62_T112 | 34.00 | 34.00 | 1 | 1 | 1 |
| T-057 | wholeblood_woman_T-057 | U133Plus2_011207W_SL36_T057 | 31.86 | 31.86 | 1 | 1 | 1 |
| T-179 | wholeblood_woman_T-179 | U133Plus2_101707W(2)_SL46_T179 | 32.14 | 34.14 | 0 | 0 | 1 |
| T-239 | wholeblood_woman_T-239 | U133Plus2_101007W_SL18_T239 | 29.71 | 35.57 | 0 | 0 | 1 |
| T-105 | wholeblood_woman_T-105 | U133Plus2_011207W_SL56_T105 | 34.86 | 34.86 | 1 | 1 | 1 |
| T-050 | wholeblood_woman_T-050 | U133Plus2_011207W_SL31_T050 | 35.00 | 35.29 | 1 | 1 | 1 |
| T-199 | wholeblood_woman_T-199 | U133Plus2_102507W(2)_SL59_T199 | 34.00 | 34.00 | 1 | 1 | 1 |
| T-261 | wholeblood_woman_T-261 | U133Plus2_103107W(2)_SL71_T261 | 25.43 | 41.29 | 0 | 0 | 0 |
| T-065 | wholeblood_woman_T-065 | U133Plus2_101707W_SL27_T065 | 32.00 | 36.00 | 0 | 0 | 1 |
| T-260 | wholeblood_woman_T-260 | U133Plus2_103107W(2)_SL70_T260 | 33.14 | 39.57 | 0 | 0 | 0 |
| T-214 | wholeblood_woman_T-214 | U133Plus2_101007W_SL02_T214 | 35.29 | 38.14 | 0 | 0 | 0 |

**Table S2. The list of 469 significant differentially expressed genes obtained using *Limma* ranked by magnitude of fold change.**

| **Entrez** | **HGNC Symbol** | **Log_2_** | **Fold Change** | **% Change** | **Average** | **adj.P.Val** | **Gene Annotation** |  |  |
| --- | --- | --- | --- | --- | --- | --- | --- | --- | --- |
| **Gene ID** |  | **Fold Change** |  |  | **Expression** | **(FDR)** |  |  |  |
| **Up-regulated genes (n=256)** | |  |  |  |  |  |  |  |  |
| 131540 | ZDHHC19 | 1.202 | 2.30 | 129.99 | 4.09 | <0.001 | zinc finger, DHHC-type containing 19 |  |  |
| 3248 | HPGD | 1.049 | 2.07 | 106.87 | 5.80 | 0.047 | hydroxyprostaglandin dehydrogenase 15-(NAD) | |  |
| 53831 | GPR84 | 0.990 | 1.99 | 98.59 | 7.22 | 0.007 | G protein-coupled receptor 84 |  |  |
| 26873 | OPLAH | 0.934 | 1.91 | 91.00 | 6.27 | 0.003 | 5-oxoprolinase (ATP-hydrolysing) |  |  |
| 122402 | TDRD9 | 0.906 | 1.87 | 87.34 | 4.43 | 0.018 | tudor domain containing 9 |  |  |
| 10079 | ATP9A | 0.880 | 1.84 | 84.10 | 7.25 | 0.013 | ATPase, class II, type 9A |  |  |
| 79623 | GALNT14 | 0.879 | 1.84 | 83.89 | 8.47 | <0.001 | UDP-N-acetyl-alpha-D-galactosamine:polypeptide N-acetylgalactosaminyltransferase 14 (GalNAc-T14) | | |
| 116369 | SLC26A8 | 0.802 | 1.74 | 74.35 | 6.90 | 0.015 | solute carrier family 26, member 8 |  |  |
| 55755 | CDK5RAP2 | 0.768 | 1.70 | 70.34 | 8.41 | 0.017 | CDK5 regulatory subunit associated protein 2 | |  |
| 399972 | ST3GAL4-AS1 | 0.760 | 1.69 | 69.37 | 8.60 | 0.015 | ST3GAL4 antisense RNA 1 (head to head) |  |  |
| 50486 | G0S2 | 0.758 | 1.69 | 69.08 | 7.11 | 0.006 | G0/G1switch 2 |  |  |
| 9021 | SOCS3 | 0.757 | 1.69 | 68.96 | 7.96 | 0.003 | suppressor of cytokine signaling 3 |  |  |
| 55647 | RAB20 | 0.711 | 1.64 | 63.72 | 8.42 | 0.009 | RAB20, member RAS oncogene family |  |  |
| 6484 | ST3GAL4 | 0.709 | 1.63 | 63.42 | 6.12 | 0.003 | ST3 beta-galactoside alpha-2,3-sialyltransferase 4 | |  |
| 199675 | C19orf59 | 0.701 | 1.63 | 62.62 | 12.28 | 0.005 | chromosome 19 open reading frame 59 |  |  |
| 2015 | EMR1 | 0.699 | 1.62 | 62.29 | 8.74 | 0.025 | egf-like module containing, mucin-like, hormone receptor-like 1 | | |
| 5209 | PFKFB3 | 0.685 | 1.61 | 60.74 | 11.06 | 0.012 | 6-phosphofructo-2-kinase/fructose-2,6-biphosphatase 3 | | |
| 731424 | LOC731424 | 0.683 | 1.61 | 60.51 | 8.60 | 0.045 | uncharacterized LOC731424 |  |  |
| 57699 | CPNE5 | 0.654 | 1.57 | 57.40 | 7.65 | 0.023 | copine V |  |  |
| 114548 | NLRP3 | 0.654 | 1.57 | 57.36 | 4.55 | 0.015 | NLR family, pyrin domain containing 3 |  |  |
| 51776 | ZAK | 0.654 | 1.57 | 57.35 | 7.57 | 0.002 | sterile alpha motif and leucine zipper containing kinase AZK | | |
| 100288432 | IL10RB-AS1 | 0.649 | 1.57 | 56.86 | 6.75 | 0.017 | IL10RB antisense RNA 1 (head to head) |  |  |
| 57089 | ENTPD7 | 0.644 | 1.56 | 56.24 | 3.06 | 0.006 | ectonucleoside triphosphate diphosphohydrolase 7 | |  |
| 92162 | TMEM88 | 0.638 | 1.56 | 55.65 | 4.96 | 0.012 | transmembrane protein 88 |  |  |
| 5447 | POR | 0.634 | 1.55 | 55.15 | 8.93 | 0.008 | P450 (cytochrome) oxidoreductase |  |  |
| 5990 | RFX2 | 0.620 | 1.54 | 53.67 | 7.40 | 0.037 | regulatory factor X, 2 (influences HLA class II expression) | | |
| 116844 | LRG1 | 0.617 | 1.53 | 53.34 | 11.51 | 0.013 | leucine-rich alpha-2-glycoprotein 1 |  |  |
| 266747 | RGL4 | 0.612 | 1.53 | 52.87 | 9.83 | 0.018 | ral guanine nucleotide dissociation stimulator-like 4 | |  |
| 84034 | EMILIN2 | 0.611 | 1.53 | 52.77 | 6.84 | 0.015 | elastin microfibril interfacer 2 |  |  |
| 11138 | TBC1D8 | 0.605 | 1.52 | 52.08 | 7.96 | 0.030 | TBC1 domain family, member 8 (with GRAM domain) | |  |
| 1230 | CCR1 | 0.605 | 1.52 | 52.07 | 11.07 | 0.022 | chemokine (C-C motif) receptor 1 |  |  |
| 10865 | ARID5A | 0.604 | 1.52 | 52.04 | 8.09 | 0.004 | AT rich interactive domain 5A (MRF1-like) |  |  |
| 151056 | PLB1 | 0.598 | 1.51 | 51.40 | 7.93 | 0.004 | phospholipase B1 |  |  |
| 2355 | FOSL2 | 0.593 | 1.51 | 50.88 | 7.16 | 0.021 | FOS-like antigen 2 |  |  |
| 5055 | SERPINB2 | 0.592 | 1.51 | 50.75 | 3.33 | 0.034 | serpin peptidase inhibitor, clade B (ovalbumin), member 2 | | |
| 7378 | UPP1 | 0.586 | 1.50 | 50.15 | 10.30 | 0.006 | uridine phosphorylase 1 |  |  |
| 5008 | OSM | 0.584 | 1.50 | 49.93 | 6.15 | 0.039 | oncostatin M |  |  |
| 23564 | DDAH2 | 0.579 | 1.49 | 49.41 | 8.20 | 0.003 | dimethylarginine dimethylaminohydrolase 2 | |  |
| 83853 | ROPN1L | 0.577 | 1.49 | 49.22 | 9.18 | 0.038 | rhophilin associated tail protein 1-like |  |  |
| 64651 | CSRNP1 | 0.576 | 1.49 | 49.10 | 9.35 | 0.014 | cysteine-serine-rich nuclear protein 1 |  |  |
| 5768 | QSOX1 | 0.576 | 1.49 | 49.10 | 7.49 | 0.023 | quiescin Q6 sulfhydryl oxidase 1 |  |  |
| 9605 | VPS9D1 | 0.571 | 1.49 | 48.57 | 7.09 | 0.003 | VPS9 domain containing 1 |  |  |
| 100506119 | LOC100506119 | 0.571 | 1.49 | 48.52 | 5.95 | 0.018 | uncharacterized LOC100506119 |  |  |
| 54512 | EXOSC4 | 0.570 | 1.48 | 48.42 | 7.57 | 0.002 | exosome component 4 |  |  |
| 222487 | GPR97 | 0.569 | 1.48 | 48.36 | 11.04 | 0.025 | G protein-coupled receptor 97 |  |  |
| 147015 | DHRS13 | 0.562 | 1.48 | 47.59 | 10.29 | 0.006 | dehydrogenase/reductase (SDR family) member 13 | |  |
| 1263 | PLK3 | 0.550 | 1.46 | 46.44 | 6.65 | 0.012 | polo-like kinase 3 |  |  |
| 100506115 | LOC100506115 | 0.541 | 1.45 | 45.48 | 11.60 | 0.022 | uncharacterized LOC100506115 |  |  |
| 8870 | IER3 | 0.532 | 1.45 | 44.56 | 9.18 | 0.028 | immediate early response 3 |  |  |
| 2153 | F5 | 0.531 | 1.45 | 44.52 | 8.02 | 0.032 | coagulation factor V (proaccelerin, labile factor) | |  |
| 4016 | LOXL1 | 0.528 | 1.44 | 44.16 | 4.08 | 0.018 | lysyl oxidase-like 1 |  |  |
| 79650 | USB1 | 0.521 | 1.43 | 43.50 | 9.19 | 0.003 | U6 snRNA biogenesis 1 |  |  |
| 3101 | HK3 | 0.521 | 1.43 | 43.50 | 10.36 | 0.003 | hexokinase 3 (white cell) |  |  |
| 200931 | SLC51A | 0.517 | 1.43 | 43.12 | 2.63 | 0.036 | solute carrier family 51, alpha subunit |  |  |
| 79865 | TREML2 | 0.514 | 1.43 | 42.77 | 9.54 | 0.037 | triggering receptor expressed on myeloid cells-like 2 | |  |
| 7100 | TLR5 | 0.512 | 1.43 | 42.59 | 10.68 | 0.013 | toll-like receptor 5 |  |  |
| 55129 | ANO10 | 0.504 | 1.42 | 41.84 | 7.46 | 0.019 | anoctamin 10 |  |  |
| 220929 | ZNF438 | 0.495 | 1.41 | 40.96 | 7.62 | 0.045 | zinc finger protein 438 |  |  |
| 79134 | TMEM185B | 0.495 | 1.41 | 40.91 | 7.65 | 0.032 | transmembrane protein 185B |  |  |
| 2992 | GYG1 | 0.494 | 1.41 | 40.82 | 13.16 | 0.012 | glycogenin 1 |  |  |
| 23095 | KIF1B | 0.493 | 1.41 | 40.72 | 8.75 | 0.031 | kinesin family member 1B |  |  |
| 2242 | FES | 0.489 | 1.40 | 40.33 | 9.26 | 0.007 | feline sarcoma oncogene |  |  |
| 83862 | TMEM120A | 0.486 | 1.40 | 40.08 | 9.61 | 0.012 | transmembrane protein 120A |  |  |
| 57655 | GRAMD1A | 0.484 | 1.40 | 39.85 | 9.76 | 0.010 | GRAM domain containing 1A |  |  |
| 147699 | PPM1N | 0.478 | 1.39 | 39.27 | 3.28 | 0.006 | protein phosphatase, Mg2+/Mn2+ dependent, 1N (putative) | | |
| 10211 | FLOT1 | 0.473 | 1.39 | 38.82 | 11.56 | 0.003 | flotillin 1 |  |  |
| 84649 | DGAT2 | 0.466 | 1.38 | 38.17 | 11.49 | 0.015 | diacylglycerol O-acyltransferase 2 |  |  |
| 6253 | RTN2 | 0.466 | 1.38 | 38.14 | 5.55 | 0.017 | reticulon 2 |  |  |
| 2131 | EXT1 | 0.464 | 1.38 | 37.97 | 7.95 | 0.019 | exostosin 1 |  |  |
| 3732 | CD82 | 0.461 | 1.38 | 37.62 | 9.96 | 0.003 | CD82 molecule |  |  |
| 9435 | CHST2 | 0.453 | 1.37 | 36.89 | 8.91 | 0.026 | carbohydrate (N-acetylglucosamine-6-O) sulfotransferase 2 | | |
| 8291 | DYSF | 0.448 | 1.36 | 36.40 | 12.77 | 0.014 | dysferlin, limb girdle muscular dystrophy 2B (autosomal recessive) | | |
| 8530 | CST7 | 0.447 | 1.36 | 36.37 | 13.49 | 0.019 | cystatin F (leukocystatin) |  |  |
| 960 | CD44 | 0.447 | 1.36 | 36.31 | 10.65 | 0.005 | CD44 molecule (Indian blood group) |  |  |
| 10435 | CDC42EP2 | 0.446 | 1.36 | 36.22 | 6.51 | 0.017 | CDC42 effector protein (Rho GTPase binding) 2 | |  |
| 9895 | TECPR2 | 0.439 | 1.36 | 35.61 | 9.25 | 0.021 | tectonin beta-propeller repeat containing 2 |  |  |
| 2319 | FLOT2 | 0.435 | 1.35 | 35.17 | 11.28 | 0.015 | flotillin 2 |  |  |
| 22926 | ATF6 | 0.427 | 1.34 | 34.46 | 10.19 | 0.015 | activating transcription factor 6 |  |  |
| 3985 | LIMK2 | 0.425 | 1.34 | 34.25 | 10.66 | 0.023 | LIM domain kinase 2 |  |  |
| 84418 | CYSTM1 | 0.418 | 1.34 | 33.60 | 13.22 | 0.017 | cysteine-rich transmembrane module containing 1 | |  |
| 27342 | RABGEF1 | 0.413 | 1.33 | 33.18 | 9.37 | 0.037 | RAB guanine nucleotide exchange factor (GEF) 1 | |  |
| 100506550 |  | 0.399 | 1.32 | 31.90 | 7.16 | 0.020 |  |  |  |
| 84925 | DIRC2 | 0.398 | 1.32 | 31.77 | 6.90 | 0.023 | disrupted in renal carcinoma 2 |  |  |
| 7090 | TLE3 | 0.398 | 1.32 | 31.76 | 9.75 | 0.036 | transducin-like enhancer of split 3 (E(sp1) homolog, Drosophila) | | |
| 3566 | IL4R | 0.398 | 1.32 | 31.73 | 10.14 | 0.029 | interleukin 4 receptor |  |  |
| 3640 | INSL3 | 0.396 | 1.32 | 31.57 | 4.66 | 0.003 | insulin-like 3 (Leydig cell) |  |  |
| 3687 | ITGAX | 0.394 | 1.31 | 31.39 | 11.49 | 0.018 | integrin, alpha X (complement component 3 receptor 4 subunit) | | |
| 3553 | IL1B | 0.393 | 1.31 | 31.33 | 10.25 | 0.044 | interleukin 1, beta |  |  |
| 51317 | PHF21A | 0.393 | 1.31 | 31.32 | 8.82 | 0.028 | PHD finger protein 21A |  |  |
| 10162 | LPCAT3 | 0.389 | 1.31 | 30.97 | 6.81 | 0.037 | lysophosphatidylcholine acyltransferase 3 |  |  |
| 27180 | SIGLEC9 | 0.388 | 1.31 | 30.84 | 7.89 | 0.046 | sialic acid binding Ig-like lectin 9 |  |  |
| 10226 | PLIN3 | 0.387 | 1.31 | 30.73 | 10.58 | 0.027 | perilipin 3 |  |  |
| 9489 | PGS1 | 0.386 | 1.31 | 30.72 | 11.28 | 0.039 | phosphatidylglycerophosphate synthase 1 | |  |
| 3726 | JUNB | 0.386 | 1.31 | 30.65 | 10.72 | 0.013 | jun B proto-oncogene |  |  |
| 8563 | THOC5 | 0.375 | 1.30 | 29.65 | 9.83 | 0.015 | THO complex 5 |  |  |
| 10538 | BATF | 0.373 | 1.30 | 29.51 | 8.27 | 0.005 | basic leucine zipper transcription factor, ATF-like | |  |
| 4043 | LRPAP1 | 0.373 | 1.29 | 29.47 | 9.89 | 0.015 | low density lipoprotein receptor-related protein associated protein 1 | | |
| 24144 | TFIP11 | 0.372 | 1.29 | 29.38 | 9.07 | 0.032 | tuftelin interacting protein 11 |  |  |
| 26502 | NARF | 0.371 | 1.29 | 29.34 | 11.43 | 0.003 | nuclear prelamin A recognition factor |  |  |
| 10603 | SH2B2 | 0.369 | 1.29 | 29.14 | 9.86 | 0.048 | SH2B adaptor protein 2 |  |  |
| 148479 | PHF13 | 0.366 | 1.29 | 28.85 | 6.66 | 0.048 | PHD finger protein 13 |  |  |
| 90007 | MIDN | 0.364 | 1.29 | 28.70 | 5.76 | 0.025 | midnolin |  |  |
| 377 | ARF3 | 0.364 | 1.29 | 28.69 | 9.32 | 0.015 | ADP-ribosylation factor 3 |  |  |
| 84674 | CARD6 | 0.364 | 1.29 | 28.66 | 10.53 | 0.023 | caspase recruitment domain family, member 6 | |  |
| 126014 | OSCAR | 0.363 | 1.29 | 28.64 | 9.47 | 0.046 | osteoclast associated, immunoglobulin-like receptor | |  |
| 56996 | SLC12A9 | 0.363 | 1.29 | 28.62 | 7.17 | 0.015 | solute carrier family 12, member 9 |  |  |
| 90459 | ERI1 | 0.360 | 1.28 | 28.37 | 6.91 | 0.045 | exoribonuclease 1 |  |  |
| 60685 | ZFAND3 | 0.358 | 1.28 | 28.13 | 7.87 | 0.039 | zinc finger, AN1-type domain 3 |  |  |
| 10981 | RAB32 | 0.357 | 1.28 | 28.05 | 10.85 | 0.023 | RAB32, member RAS oncogene family |  |  |
| 64778 | FNDC3B | 0.357 | 1.28 | 28.04 | 9.22 | 0.041 | fibronectin type III domain containing 3B |  |  |
| 55784 | MCTP2 | 0.356 | 1.28 | 27.98 | 8.69 | 0.049 | multiple C2 domains, transmembrane 2 |  |  |
| 3718 | JAK3 | 0.355 | 1.28 | 27.93 | 5.84 | <0.001 | Janus kinase 3 |  |  |
| 1889 | ECE1 | 0.354 | 1.28 | 27.79 | 7.49 | 0.022 | endothelin converting enzyme 1 |  |  |
| 5130 | PCYT1A | 0.353 | 1.28 | 27.74 | 5.94 | 0.030 | phosphate cytidylyltransferase 1, choline, alpha | |  |
| 4298 | MLLT1 | 0.353 | 1.28 | 27.71 | 7.38 | 0.003 | myeloid/lymphoid or mixed-lineage leukemia (trithorax homolog, Drosophila); translocated to, 1 | | |
| 284207 | METRNL | 0.352 | 1.28 | 27.59 | 9.49 | 0.036 | meteorin, glial cell differentiation regulator-like | |  |
| 113878 | DTX2 | 0.350 | 1.27 | 27.47 | 6.25 | 0.032 | deltex homolog 2 (Drosophila) |  |  |
| 3454 | IFNAR1 | 0.347 | 1.27 | 27.23 | 9.01 | 0.029 | interferon (alpha, beta and omega) receptor 1 | |  |
| 54432 | YIPF1 | 0.346 | 1.27 | 27.11 | 9.87 | 0.013 | Yip1 domain family, member 1 |  |  |
| 126208 | ZNF787 | 0.346 | 1.27 | 27.06 | 7.24 | 0.047 | zinc finger protein 787 |  |  |
| 1992 | SERPINB1 | 0.344 | 1.27 | 26.97 | 13.09 | 0.026 | serpin peptidase inhibitor, clade B (ovalbumin), member 1 | | |
| 632 | BGLAP | 0.343 | 1.27 | 26.88 | 3.94 | 0.029 | bone gamma-carboxyglutamate (gla) protein | |  |
| 83667 | SESN2 | 0.342 | 1.27 | 26.78 | 5.48 | 0.048 | sestrin 2 |  |  |
| 56063 | TMEM234 | 0.340 | 1.27 | 26.59 | 6.05 | 0.038 | transmembrane protein 234 |  |  |
| 283537 | SLC46A3 | 0.340 | 1.27 | 26.58 | 9.54 | 0.014 | solute carrier family 46, member 3 |  |  |
| 54187 | NANS | 0.339 | 1.26 | 26.47 | 9.09 | 0.012 | N-acetylneuraminic acid synthase |  |  |
| 23144 | ZC3H3 | 0.338 | 1.26 | 26.38 | 5.73 | 0.023 | zinc finger CCCH-type containing 3 |  |  |
| 706 | TSPO | 0.337 | 1.26 | 26.31 | 13.24 | 0.004 | translocator protein (18kDa) |  |  |
| 9674 | KIAA0040 | 0.337 | 1.26 | 26.27 | 9.66 | 0.045 | KIAA0040 |  |  |
| 2584 | GALK1 | 0.336 | 1.26 | 26.18 | 5.97 | 0.039 | galactokinase 1 |  |  |
| 132160 | PPM1M | 0.335 | 1.26 | 26.12 | 10.11 | 0.005 | protein phosphatase, Mg2+/Mn2+ dependent, 1M | |  |
| 2800 | GOLGA1 | 0.332 | 1.26 | 25.92 | 5.64 | 0.045 | golgin A1 |  |  |
| 4296 | MAP3K11 | 0.332 | 1.26 | 25.85 | 8.84 | 0.041 | mitogen-activated protein kinase kinase kinase 11 | |  |
| 6195 | RPS6KA1 | 0.332 | 1.26 | 25.85 | 11.09 | 0.014 | ribosomal protein S6 kinase, 90kDa, polypeptide 1 | |  |
| 23534 | TNPO3 | 0.331 | 1.26 | 25.78 | 9.07 | 0.048 | transportin 3 |  |  |
| 3684 | ITGAM | 0.329 | 1.26 | 25.61 | 12.31 | 0.013 | integrin, alpha M (complement component 3 receptor 3 subunit) | | |
| 493 | ATP2B4 | 0.328 | 1.26 | 25.54 | 8.61 | 0.007 | ATPase, Ca++ transporting, plasma membrane 4 | |  |
| 7076 | TIMP1 | 0.328 | 1.26 | 25.51 | 12.08 | 0.042 | TIMP metallopeptidase inhibitor 1 |  |  |
| 8694 | DGAT1 | 0.327 | 1.25 | 25.43 | 8.24 | 0.008 | diacylglycerol O-acyltransferase 1 |  |  |
| 51271 | UBAP1 | 0.326 | 1.25 | 25.32 | 9.36 | 0.023 | ubiquitin associated protein 1 |  |  |
| 51296 | SLC15A3 | 0.325 | 1.25 | 25.26 | 9.30 | 0.033 | solute carrier family 15 (oligopeptide transporter), member 3 | | |
| 28988 | DBNL | 0.324 | 1.25 | 25.18 | 9.49 | 0.026 | drebrin-like |  |  |
| 3516 | RBPJ | 0.323 | 1.25 | 25.09 | 12.68 | 0.015 | recombination signal binding protein for immunoglobulin kappa J region | | |
| 64319 | FBRS | 0.320 | 1.25 | 24.83 | 6.97 | 0.049 | fibrosin |  |  |
| 1084 | CEACAM3 | 0.320 | 1.25 | 24.81 | 9.14 | 0.032 | carcinoembryonic antigen-related cell adhesion molecule 3 | | |
| 3577 | CXCR1 | 0.320 | 1.25 | 24.81 | 12.96 | 0.021 | chemokine (C-X-C motif) receptor 1 |  |  |
| 10295 | BCKDK | 0.319 | 1.25 | 24.79 | 8.37 | 0.022 | branched chain ketoacid dehydrogenase kinase | |  |
| 11025 | LILRB3 | 0.319 | 1.25 | 24.78 | 12.03 | 0.016 | leukocyte immunoglobulin-like receptor, subfamily B (with TM and ITIM domains), member 3 | | |
| 254102 | EHBP1L1 | 0.319 | 1.25 | 24.72 | 8.51 | 0.017 | EH domain binding protein 1-like 1 |  |  |
| 9929 | JOSD1 | 0.317 | 1.25 | 24.54 | 8.50 | 0.013 | Josephin domain containing 1 |  |  |
| 80301 | PLEKHO2 | 0.316 | 1.24 | 24.50 | 10.98 | 0.013 | pleckstrin homology domain containing, family O member 2 | | |
| 3280 | HES1 | 0.315 | 1.24 | 24.38 | 3.61 | 0.022 | hes family bHLH transcription factor 1 |  |  |
| 6483 | ST3GAL2 | 0.315 | 1.24 | 24.37 | 7.84 | 0.030 | ST3 beta-galactoside alpha-2,3-sialyltransferase 2 | |  |
| 27128 | CYTH4 | 0.312 | 1.24 | 24.16 | 9.33 | 0.035 | cytohesin 4 |  |  |
| 28232 | SLCO3A1 | 0.311 | 1.24 | 24.06 | 8.47 | 0.041 | solute carrier organic anion transporter family, member 3A1 | | |
| 3383 | ICAM1 | 0.309 | 1.24 | 23.86 | 7.13 | 0.044 | intercellular adhesion molecule 1 |  |  |
| 7132 | TNFRSF1A | 0.308 | 1.24 | 23.83 | 12.62 | 0.023 | tumor necrosis factor receptor superfamily, member 1A | |  |
| 2590 | GALNT2 | 0.306 | 1.24 | 23.67 | 7.09 | 0.012 | UDP-N-acetyl-alpha-D-galactosamine:polypeptide N-acetylgalactosaminyltransferase 2 (GalNAc-T2) | | |
| 642475 | MROH6 | 0.306 | 1.24 | 23.60 | 5.34 | 0.003 | maestro heat-like repeat family member 6 |  |  |
| 2787 | GNG5 | 0.305 | 1.24 | 23.50 | 11.44 | 0.037 | guanine nucleotide binding protein (G protein), gamma 5 | | |
| 55902 | ACSS2 | 0.300 | 1.23 | 23.11 | 7.20 | 0.015 | acyl-CoA synthetase short-chain family member 2 | |  |
| 10938 | EHD1 | 0.298 | 1.23 | 22.94 | 9.74 | 0.015 | EH-domain containing 1 |  |  |
| 967 | CD63 | 0.298 | 1.23 | 22.94 | 13.13 | 0.006 | CD63 molecule |  |  |
| 6515 | SLC2A3 | 0.298 | 1.23 | 22.91 | 12.39 | 0.035 | solute carrier family 2 (facilitated glucose transporter), member 3 | | |
| 5770 | PTPN1 | 0.296 | 1.23 | 22.75 | 7.84 | 0.037 | protein tyrosine phosphatase, non-receptor type 1 | |  |
| 83706 | FERMT3 | 0.294 | 1.23 | 22.58 | 10.94 | 0.027 | fermitin family member 3 |  |  |
| 58986 | TMEM8A | 0.294 | 1.23 | 22.58 | 8.24 | 0.041 | transmembrane protein 8A |  |  |
| 83463 | MXD3 | 0.294 | 1.23 | 22.58 | 8.73 | 0.031 | MAX dimerization protein 3 |  |  |
| 200058 | FLJ23867 | 0.293 | 1.23 | 22.53 | 3.34 | 0.045 | uncharacterized protein FLJ23867 |  |  |
| 79415 | C17orf62 | 0.293 | 1.23 | 22.52 | 12.02 | 0.005 | chromosome 17 open reading frame 62 |  |  |
| 58500 | ZNF250 | 0.292 | 1.22 | 22.43 | 2.60 | 0.013 | zinc finger protein 250 |  |  |
| 100131117 | ZBTB20-AS1 | 0.291 | 1.22 | 22.34 | 2.68 | 0.036 | ZBTB20 antisense RNA 1 |  |  |
| 100290566 |  | 0.290 | 1.22 | 22.27 | 8.06 | 0.020 |  |  |  |
| 10049 | DNAJB6 | 0.290 | 1.22 | 22.23 | 9.04 | 0.034 | DnaJ (Hsp40) homolog, subfamily B, member 6 | |  |
| 4790 | NFKB1 | 0.289 | 1.22 | 22.18 | 9.76 | 0.037 | nuclear factor of kappa light polypeptide gene enhancer in B-cells 1 | | |
| 9673 | SLC25A44 | 0.288 | 1.22 | 22.11 | 10.26 | 0.047 | solute carrier family 25, member 44 |  |  |
| 9683 | N4BP1 | 0.286 | 1.22 | 21.89 | 9.07 | 0.029 | NEDD4 binding protein 1 |  |  |
| 124583 | CANT1 | 0.282 | 1.22 | 21.56 | 9.80 | 0.048 | calcium activated nucleotidase 1 |  |  |
| 7409 | VAV1 | 0.278 | 1.21 | 21.23 | 10.62 | 0.021 | vav 1 guanine nucleotide exchange factor |  |  |
| 54434 | SSH1 | 0.278 | 1.21 | 21.22 | 4.81 | 0.030 | slingshot protein phosphatase 1 |  |  |
| 1523 | CUX1 | 0.278 | 1.21 | 21.21 | 8.86 | 0.021 | cut-like homeobox 1 |  |  |
| 2207 | FCER1G | 0.277 | 1.21 | 21.17 | 14.24 | 0.003 | Fc fragment of IgE, high affinity I, receptor for; gamma polypeptide | | |
| 415116 | PIM3 | 0.275 | 1.21 | 20.97 | 10.59 | 0.019 | pim-3 oncogene |  |  |
| 3055 | HCK | 0.275 | 1.21 | 20.97 | 13.95 | 0.026 | hemopoietic cell kinase |  |  |
| 64374 | SIL1 | 0.274 | 1.21 | 20.91 | 5.70 | 0.036 | SIL1 nucleotide exchange factor |  |  |
| 9961 | MVP | 0.271 | 1.21 | 20.66 | 11.17 | 0.030 | major vault protein |  |  |
| 23307 | FKBP15 | 0.271 | 1.21 | 20.63 | 9.19 | 0.032 | FK506 binding protein 15, 133kDa |  |  |
| 10011 | SRA1 | 0.271 | 1.21 | 20.62 | 10.00 | 0.022 | steroid receptor RNA activator 1 |  |  |
| 9654 | TTLL4 | 0.263 | 1.20 | 19.97 | 4.04 | 0.015 | tubulin tyrosine ligase-like family, member 4 | |  |
| 391 | RHOG | 0.261 | 1.20 | 19.81 | 13.64 | 0.020 | ras homolog family member G |  |  |
| 55737 | VPS35 | 0.260 | 1.20 | 19.78 | 10.99 | 0.014 | vacuolar protein sorting 35 homolog (S. cerevisiae) | |  |
| 3588 | IL10RB | 0.259 | 1.20 | 19.68 | 12.02 | 0.045 | interleukin 10 receptor, beta |  |  |
| 23210 | JMJD6 | 0.258 | 1.20 | 19.61 | 6.78 | 0.031 | jumonji domain containing 6 |  |  |
| 7920 | ABHD16A | 0.258 | 1.20 | 19.59 | 9.27 | 0.020 | abhydrolase domain containing 16A |  |  |
| 51292 | GMPR2 | 0.257 | 1.19 | 19.46 | 10.00 | 0.037 | guanosine monophosphate reductase 2 |  |  |
| 4055 | LTBR | 0.252 | 1.19 | 19.12 | 7.80 | 0.015 | lymphotoxin beta receptor (TNFR superfamily, member 3) | | |
| 7227 | TRPS1 | 0.252 | 1.19 | 19.06 | 4.84 | 0.047 | trichorhinophalangeal syndrome I |  |  |
| 55741 | EDEM2 | 0.250 | 1.19 | 18.92 | 9.34 | 0.023 | ER degradation enhancer, mannosidase alpha-like 2 | |  |
| 64342 | HS1BP3 | 0.249 | 1.19 | 18.80 | 8.67 | 0.046 | HCLS1 binding protein 3 |  |  |
| 8793 | TNFRSF10D | 0.248 | 1.19 | 18.76 | 3.79 | 0.026 | tumor necrosis factor receptor superfamily, member 10d, decoy with truncated death domain | | |
| 29950 | SERTAD1 | 0.248 | 1.19 | 18.72 | 8.21 | 0.042 | SERTA domain containing 1 |  |  |
| 57053 | CHRNA10 | 0.247 | 1.19 | 18.71 | 2.56 | 0.015 | cholinergic receptor, nicotinic, alpha 10 (neuronal) | |  |
| 11270 | NRM | 0.247 | 1.19 | 18.68 | 8.03 | 0.035 | nurim (nuclear envelope membrane protein) | |  |
| 81894 | SLC25A28 | 0.245 | 1.18 | 18.48 | 8.26 | 0.037 | solute carrier family 25 (mitochondrial iron transporter), member 28 | | |
| 2870 | GRK6 | 0.243 | 1.18 | 18.34 | 9.76 | 0.018 | G protein-coupled receptor kinase 6 |  |  |
| 79180 | EFHD2 | 0.243 | 1.18 | 18.33 | 11.51 | 0.041 | EF-hand domain family, member D2 |  |  |
| 83692 | CD99L2 | 0.243 | 1.18 | 18.32 | 4.64 | 0.029 | CD99 molecule-like 2 |  |  |
| 9922 | IQSEC1 | 0.241 | 1.18 | 18.16 | 10.69 | 0.023 | IQ motif and Sec7 domain 1 |  |  |
| 54926 | UBE2R2 | 0.240 | 1.18 | 18.10 | 11.02 | 0.045 | ubiquitin-conjugating enzyme E2R 2 |  |  |
| 51304 | ZDHHC3 | 0.239 | 1.18 | 18.05 | 7.66 | 0.022 | zinc finger, DHHC-type containing 3 |  |  |
| 6648 | SOD2 | 0.235 | 1.18 | 17.73 | 13.41 | 0.047 | superoxide dismutase 2, mitochondrial |  |  |
| 5265 | SERPINA1 | 0.234 | 1.18 | 17.61 | 14.55 | 0.014 | serpin peptidase inhibitor, clade A (alpha-1 antiproteinase, antitrypsin), member 1 | | |
| 11035 | RIPK3 | 0.232 | 1.17 | 17.41 | 7.86 | 0.027 | receptor-interacting serine-threonine kinase 3 | |  |
| 4689 | NCF4 | 0.231 | 1.17 | 17.35 | 13.11 | 0.037 | neutrophil cytosolic factor 4, 40kDa |  |  |
| 7177 | TPSAB1 | 0.227 | 1.17 | 17.00 | 6.63 | 0.035 | tryptase alpha/beta 1 |  |  |
| 404201 | WDFY3-AS2 | 0.223 | 1.17 | 16.75 | 2.29 | 0.001 | WDFY3 antisense RNA 2 |  |  |
| 10409 | BASP1 | 0.221 | 1.17 | 16.57 | 14.68 | 0.023 | brain abundant, membrane attached signal protein 1 | |  |
| 22877 | MLXIP | 0.221 | 1.17 | 16.56 | 5.32 | 0.049 | MLX interacting protein |  |  |
| 553115 | PEF1 | 0.218 | 1.16 | 16.28 | 9.84 | 0.012 | penta-EF-hand domain containing 1 |  |  |
| 5937 | RBMS1 | 0.217 | 1.16 | 16.21 | 11.35 | 0.020 | RNA binding motif, single stranded interacting protein 1 | | |
| 55654 | TMEM127 | 0.215 | 1.16 | 16.06 | 9.56 | 0.047 | transmembrane protein 127 |  |  |
| 54838 | WBP1L | 0.213 | 1.16 | 15.88 | 9.91 | 0.046 | WW domain binding protein 1-like |  |  |
| 25923 | ATL3 | 0.208 | 1.16 | 15.53 | 2.54 | 0.016 | atlastin GTPase 3 |  |  |
| 7846 | TUBA1A | 0.207 | 1.15 | 15.42 | 14.34 | 0.022 | tubulin, alpha 1a |  |  |
| 58190 | CTDSP1 | 0.205 | 1.15 | 15.28 | 10.38 | 0.042 | CTD (carboxy-terminal domain, RNA polymerase II, polypeptide A) small phosphatase 1 | | |
| 79709 | COLGALT1 | 0.196 | 1.15 | 14.52 | 6.06 | 0.034 | collagen beta(1-O)galactosyltransferase 1 |  |  |
| 79594 | MUL1 | 0.195 | 1.14 | 14.45 | 6.13 | 0.047 | mitochondrial E3 ubiquitin protein ligase 1 |  |  |
| 7994 | KAT6A | 0.191 | 1.14 | 14.19 | 10.43 | 0.046 | K(lysine) acetyltransferase 6A |  |  |
| 80347 | COASY | 0.189 | 1.14 | 14.00 | 8.17 | 0.032 | CoA synthase |  |  |
| 6821 | SUOX | 0.186 | 1.14 | 13.80 | 3.36 | 0.030 | sulfite oxidase |  |  |
| 226 | ALDOA | 0.186 | 1.14 | 13.79 | 12.58 | 0.041 | aldolase A, fructose-bisphosphate |  |  |
| 26277 | TINF2 | 0.180 | 1.13 | 13.33 | 10.51 | 0.010 | TERF1 (TRF1)-interacting nuclear factor 2 |  |  |
| 7038 | TG | 0.174 | 1.13 | 12.79 | 2.34 | 0.044 | thyroglobulin |  |  |
| 4898 | NRD1 | 0.173 | 1.13 | 12.71 | 11.97 | 0.050 | nardilysin (N-arginine dibasic convertase) |  |  |
| 10221 | TRIB1 | 0.173 | 1.13 | 12.71 | 2.45 | 0.041 | tribbles pseudokinase 1 |  |  |
| 79155 | TNIP2 | 0.171 | 1.13 | 12.56 | 8.87 | 0.026 | TNFAIP3 interacting protein 2 |  |  |
| 7084 | TK2 | 0.166 | 1.12 | 12.22 | 4.85 | 0.022 | thymidine kinase 2, mitochondrial |  |  |
| 8239 | USP9X | 0.161 | 1.12 | 11.84 | 9.32 | 0.037 | ubiquitin specific peptidase 9, X-linked |  |  |
| 2582 | GALE | 0.144 | 1.10 | 10.48 | 2.50 | 0.028 | UDP-galactose-4-epimerase |  |  |
| 23288 | IQCE | 0.140 | 1.10 | 10.23 | 3.59 | 0.044 | IQ motif containing E |  |  |
| 351 | APP | 0.130 | 1.09 | 9.39 | 6.14 | 0.030 | amyloid beta (A4) precursor protein |  |  |
| 10540 | DCTN2 | 0.127 | 1.09 | 9.22 | 10.18 | 0.047 | dynactin 2 (p50) |  |  |
| 6282 | S100A11 | 0.126 | 1.09 | 9.12 | 15.13 | 0.029 | S100 calcium binding protein A11 |  |  |
| 63924 | CIDEC | 0.125 | 1.09 | 9.03 | 2.27 | 0.016 | cell death-inducing DFFA-like effector c |  |  |
| 10018 | BCL2L11 | 0.123 | 1.09 | 8.90 | 5.36 | 0.047 | BCL2-like 11 (apoptosis facilitator) |  |  |
| 2810 | SFN | 0.115 | 1.08 | 8.30 | 3.08 | 0.037 | stratifin |  |  |
| 116986 | AGAP2 | 0.113 | 1.08 | 8.18 | 2.36 | 0.014 | ArfGAP with GTPase domain, ankyrin repeat and PH domain 2 | | |
| 55252 | ASXL2 | 0.106 | 1.08 | 7.64 | 3.28 | 0.038 | additional sex combs like 2 (Drosophila) |  |  |
| 711 | ERC2-IT1 | 0.093 | 1.07 | 6.69 | 2.30 | 0.037 | ERC2 intronic transcript 1 (non-protein coding) | |  |
| 5830 | PEX5 | 0.082 | 1.06 | 5.87 | 2.96 | 0.029 | peroxisomal biogenesis factor 5 |  |  |
| 389792 | IER5L | 0.078 | 1.06 | 5.58 | 3.64 | 0.026 | immediate early response 5-like |  |  |
| 7170 | TPM3 | 0.076 | 1.05 | 5.39 | 2.87 | 0.017 | tropomyosin 3 |  |  |
| 283875 | LINC00514 | 0.067 | 1.05 | 4.78 | 2.23 | 0.026 | long intergenic non-protein coding RNA 514 | |  |
| 100505547 | LOC100505547 | 0.061 | 1.04 | 4.35 | 2.24 | 0.039 | uncharacterized LOC100505547 |  |  |
| 153562 | MARVELD2 | 0.052 | 1.04 | 3.66 | 2.24 | 0.047 | MARVEL domain containing 2 |  |  |
| 6462 | SHBG | 0.027 | 1.02 | 1.86 | 2.22 | 0.023 | sex hormone-binding globulin |  |  |
| **Down-regulated genes (n=213)** | | | | | | | | | |
| 92342 | METTL18 | -0.909 | 0.53 | -46.76 | 3.96 | 0.002 | methyltransferase like 18 |  |  |
| 23015 | GOLGA8A | -0.874 | 0.55 | -45.44 | 8.94 | 0.037 | golgin A8 family, member A |  |  |
| 55900 | ZNF302 | -0.802 | 0.57 | -42.65 | 3.70 | 0.007 | zinc finger protein 302 |  |  |
| 54482 | TRMT13 | -0.797 | 0.58 | -42.46 | 4.87 | 0.047 | tRNA methyltransferase 13 homolog (S. cerevisiae) | |  |
| 1070 | CETN3 | -0.773 | 0.59 | -41.49 | 4.45 | 0.032 | centrin, EF-hand protein, 3 |  |  |
| 79752 | ZFAND1 | -0.771 | 0.59 | -41.41 | 5.87 | 0.040 | zinc finger, AN1-type domain 1 |  |  |
| 26228 | STAP1 | -0.764 | 0.59 | -41.11 | 4.61 | 0.007 | signal transducing adaptor family member 1 | |  |
| 931 | MS4A1 | -0.758 | 0.59 | -40.86 | 7.52 | 0.028 | membrane-spanning 4-domains, subfamily A, member 1 | | |
| 57560 | IFT80 | -0.737 | 0.60 | -40.01 | 4.62 | 0.040 | intraflagellar transport 80 homolog (Chlamydomonas) | |  |
| 10393 | ANAPC10 | -0.729 | 0.60 | -39.65 | 4.42 | 0.047 | anaphase promoting complex subunit 10 |  |  |
| 60592 | SCOC | -0.705 | 0.61 | -38.64 | 4.88 | 0.047 | short coiled-coil protein |  |  |
| 100499177 | THAP9-AS1 | -0.703 | 0.61 | -38.56 | 7.05 | 0.024 | THAP9 antisense RNA 1 |  |  |
| 6741 | SSB | -0.686 | 0.62 | -37.86 | 6.57 | 0.026 | Sjogren syndrome antigen B (autoantigen La) | |  |
| 157567 | ANKRD46 | -0.671 | 0.63 | -37.20 | 4.70 | 0.037 | ankyrin repeat domain 46 |  |  |
| 112942 | CCDC104 | -0.668 | 0.63 | -37.07 | 5.00 | 0.031 | coiled-coil domain containing 104 |  |  |
| 91298 | C12ORF29 | -0.648 | 0.64 | -36.17 | 4.78 | 0.031 | chromosome 12 open reading frame 29 |  |  |
| 5378 | PMS1 | -0.647 | 0.64 | -36.13 | 5.33 | 0.044 | PMS1 postmeiotic segregation increased 1 (S. cerevisiae) | | |
| 8821 | INPP4B | -0.636 | 0.64 | -35.67 | 4.85 | 0.012 | inositol polyphosphate-4-phosphatase, type II, 105kDa | | |
| 84128 | WDR75 | -0.636 | 0.64 | -35.67 | 6.72 | 0.019 | WD repeat domain 75 |  |  |
| 65109 | UPF3B | -0.626 | 0.65 | -35.18 | 4.23 | 0.031 | UPF3 regulator of nonsense transcripts homolog B (yeast) | | |
| 123036 | TC2N | -0.624 | 0.65 | -35.10 | 6.22 | 0.021 | tandem C2 domains, nuclear |  |  |
| 8504 | PEX3 | -0.620 | 0.65 | -34.93 | 4.77 | 0.021 | peroxisomal biogenesis factor 3 |  |  |
| 27075 | TSPAN13 | -0.610 | 0.66 | -34.50 | 6.75 | 0.022 | tetraspanin 13 |  |  |
| 34 | ACADM | -0.596 | 0.66 | -33.84 | 7.11 | 0.049 | acyl-CoA dehydrogenase, C-4 to C-12 straight chain | |  |
| 55300 | PI4K2B | -0.594 | 0.66 | -33.75 | 5.32 | 0.047 | phosphatidylinositol 4-kinase type 2 beta |  |  |
| 10892 | MALT1 | -0.594 | 0.66 | -33.73 | 5.88 | 0.018 | mucosa associated lymphoid tissue lymphoma translocation gene 1 | | |
| 117143 | TADA1 | -0.593 | 0.66 | -33.68 | 3.95 | 0.016 | transcriptional adaptor 1 |  |  |
| 57665 | RDH14 | -0.578 | 0.67 | -33.00 | 5.91 | 0.015 | retinol dehydrogenase 14 (all-trans/9-cis/11-cis) | |  |
| 10282 | BET1 | -0.577 | 0.67 | -32.95 | 5.95 | 0.044 | Bet1 golgi vesicular membrane trafficking protein | |  |
| 55006 | TRMT61B | -0.565 | 0.68 | -32.42 | 5.91 | 0.023 | tRNA methyltransferase 61 homolog B (S. cerevisiae) | |  |
| 84365 | NIFK | -0.562 | 0.68 | -32.25 | 6.01 | 0.030 | nucleolar protein interacting with the FHA domain of MKI67 | | |
| 25842 | ASF1A | -0.560 | 0.68 | -32.18 | 7.15 | 0.038 | anti-silencing function 1A histone chaperone | |  |
| 5567 | PRKACB | -0.559 | 0.68 | -32.11 | 6.72 | 0.032 | protein kinase, cAMP-dependent, catalytic, beta | |  |
| 9255 | AIMP1 | -0.558 | 0.68 | -32.10 | 6.61 | 0.041 | aminoacyl tRNA synthetase complex-interacting multifunctional protein 1 | | |
| 138241 | C9ORF85 | -0.554 | 0.68 | -31.89 | 3.96 | 0.031 | chromosome 9 open reading frame 85 |  |  |
| 5311 | PKD2 | -0.552 | 0.68 | -31.82 | 6.39 | 0.028 | polycystic kidney disease 2 (autosomal dominant) | |  |
| 10402 | ST3GAL6 | -0.548 | 0.68 | -31.59 | 7.37 | 0.017 | ST3 beta-galactoside alpha-2,3-sialyltransferase 6 | |  |
| 58478 | ENOPH1 | -0.546 | 0.68 | -31.51 | 7.71 | 0.020 | enolase-phosphatase 1 |  |  |
| 8725 | URI1 | -0.544 | 0.69 | -31.44 | 7.30 | 0.015 | URI1, prefoldin-like chaperone |  |  |
| 29121 | CLEC2D | -0.538 | 0.69 | -31.15 | 6.13 | 0.032 | C-type lectin domain family 2, member D |  |  |
| 81537 | SGPP1 | -0.537 | 0.69 | -31.10 | 5.52 | 0.037 | sphingosine-1-phosphate phosphatase 1 |  |  |
| 9702 | CEP57 | -0.537 | 0.69 | -31.06 | 5.97 | 0.023 | centrosomal protein 57kDa |  |  |
| 1633 | DCK | -0.536 | 0.69 | -31.03 | 8.82 | 0.039 | deoxycytidine kinase |  |  |
| 9166 | EBAG9 | -0.535 | 0.69 | -31.00 | 6.96 | 0.016 | estrogen receptor binding site associated, antigen, 9 | |  |
| 51015 | ISOC1 | -0.532 | 0.69 | -30.86 | 7.09 | 0.044 | isochorismatase domain containing 1 |  |  |
| 92912 | UBE2Q2 | -0.525 | 0.69 | -30.51 | 8.68 | 0.045 | ubiquitin-conjugating enzyme E2Q family member 2 | |  |
| 114908 | TMEM123 | -0.523 | 0.70 | -30.43 | 11.37 | 0.042 | transmembrane protein 123 |  |  |
| 23473 | CAPN7 | -0.522 | 0.70 | -30.36 | 6.08 | 0.015 | calpain 7 |  |  |
| 27334 | P2RY10 | -0.520 | 0.70 | -30.26 | 4.57 | 0.032 | purinergic receptor P2Y, G-protein coupled, 10 | |  |
| 5366 | PMAIP1 | -0.519 | 0.70 | -30.21 | 6.02 | 0.020 | phorbol-12-myristate-13-acetate-induced protein 1 | |  |
| 3646 | EIF3E | -0.517 | 0.70 | -30.12 | 11.84 | 0.045 | eukaryotic translation initiation factor 3, subunit E | |  |
| 57037 | ANKMY2 | -0.516 | 0.70 | -30.07 | 6.27 | 0.035 | ankyrin repeat and MYND domain containing 2 | |  |
| 79738 | BBS10 | -0.513 | 0.70 | -29.91 | 6.47 | 0.045 | Bardet-Biedl syndrome 10 |  |  |
| 56204 | FAM214A | -0.511 | 0.70 | -29.83 | 8.82 | 0.012 | family with sequence similarity 214, member A | |  |
| 83939 | EIF2A | -0.510 | 0.70 | -29.78 | 9.40 | 0.042 | eukaryotic translation initiation factor 2A, 65kDa | |  |
| 202781 | PAXIP1-AS1 | -0.509 | 0.70 | -29.71 | 4.29 | 0.015 | PAXIP1 antisense RNA 1 (head to head) |  |  |
| 159090 | FAM122B | -0.506 | 0.70 | -29.58 | 6.87 | 0.018 | family with sequence similarity 122B |  |  |
| 10800 | CYSLTR1 | -0.505 | 0.70 | -29.54 | 8.63 | 0.020 | cysteinyl leukotriene receptor 1 |  |  |
| 25950 | RWDD3 | -0.503 | 0.71 | -29.42 | 4.34 | 0.018 | RWD domain containing 3 |  |  |
| 9406 | ZRANB2 | -0.501 | 0.71 | -29.33 | 9.59 | 0.045 | zinc finger, RAN-binding domain containing 2 | |  |
| 60487 | TRMT11 | -0.500 | 0.71 | -29.30 | 3.47 | 0.049 | tRNA methyltransferase 11 homolog (S. cerevisiae) | |  |
| 23443 | SLC35A3 | -0.499 | 0.71 | -29.26 | 6.02 | 0.044 | solute carrier family 35 (UDP-N-acetylglucosamine (UDP-GlcNAc) transporter), member A3 | | |
| 5876 | RABGGTB | -0.498 | 0.71 | -29.19 | 5.07 | 0.020 | Rab geranylgeranyltransferase, beta subunit | |  |
| 55251 | PCMTD2 | -0.494 | 0.71 | -29.00 | 9.80 | 0.018 | protein-L-isoaspartate (D-aspartate) O-methyltransferase domain containing 2 | | |
| 51727 | CMPK1 | -0.492 | 0.71 | -28.88 | 9.80 | 0.023 | cytidine monophosphate (UMP-CMP) kinase 1, cytosolic | | |
| 2287 | FKBP3 | -0.487 | 0.71 | -28.63 | 8.63 | 0.046 | FK506 binding protein 3, 25kDa |  |  |
| 25816 | TNFAIP8 | -0.485 | 0.71 | -28.57 | 10.58 | 0.006 | tumor necrosis factor, alpha-induced protein 8 | |  |
| 7699 | ZNF140 | -0.482 | 0.72 | -28.40 | 5.80 | 0.047 | zinc finger protein 140 |  |  |
| 53371 | NUP54 | -0.481 | 0.72 | -28.37 | 8.16 | 0.046 | nucleoporin 54kDa |  |  |
| 256380 | SCML4 | -0.481 | 0.72 | -28.33 | 8.18 | 0.003 | sex comb on midleg-like 4 (Drosophila) |  |  |
| 7748 | ZNF195 | -0.477 | 0.72 | -28.18 | 3.80 | 0.019 | zinc finger protein 195 |  |  |
| 7443 | VRK1 | -0.477 | 0.72 | -28.14 | 7.52 | 0.023 | vaccinia related kinase 1 |  |  |
| 3945 | LDHB | -0.469 | 0.72 | -27.76 | 12.99 | 0.044 | lactate dehydrogenase B |  |  |
| 9338 | TCEAL1 | -0.468 | 0.72 | -27.73 | 3.40 | 0.045 | transcription elongation factor A (SII)-like 1 | |  |
| 1974 | EIF4A2 | -0.468 | 0.72 | -27.72 | 11.44 | 0.016 | eukaryotic translation initiation factor 4A2 |  |  |
| 11168 | PSIP1 | -0.467 | 0.72 | -27.65 | 8.43 | 0.015 | PC4 and SFRS1 interacting protein 1 |  |  |
| 51747 | LUC7L3 | -0.466 | 0.72 | -27.62 | 9.53 | 0.027 | LUC7-like 3 (S. cerevisiae) |  |  |
| 10772 | SRSF10 | -0.464 | 0.73 | -27.49 | 8.27 | 0.020 | serine/arginine-rich splicing factor 10 |  |  |
| 114932 | MRFAP1L1 | -0.462 | 0.73 | -27.42 | 9.05 | 0.005 | Morf4 family associated protein 1-like 1 |  |  |
| 6726 | SRP9 | -0.462 | 0.73 | -27.38 | 12.21 | 0.029 | signal recognition particle 9kDa |  |  |
| 92106 | OXNAD1 | -0.461 | 0.73 | -27.36 | 7.32 | 0.048 | oxidoreductase NAD-binding domain containing 1 | |  |
| 100506112 | RIMKLB | -0.458 | 0.73 | -27.22 | 5.00 | 0.006 | ribosomal modification protein rimK-like family member B | | |
| 91272 | BOD1 | -0.457 | 0.73 | -27.16 | 6.73 | 0.030 | biorientation of chromosomes in cell division 1 | |  |
| 7503 | XIST | -0.457 | 0.73 | -27.15 | 10.08 | 0.022 | X inactive specific transcript (non-protein coding) | |  |
| 10196 | PRMT3 | -0.456 | 0.73 | -27.10 | 4.70 | 0.047 | protein arginine methyltransferase 3 |  |  |
| 10600 | USP16 | -0.455 | 0.73 | -27.05 | 7.30 | 0.029 | ubiquitin specific peptidase 16 |  |  |
| 9847 | C2CD5 | -0.451 | 0.73 | -26.87 | 8.71 | 0.030 | C2 calcium-dependent domain containing 5 | |  |
| 50854 | C6ORF48 | -0.450 | 0.73 | -26.79 | 10.20 | 0.044 | chromosome 6 open reading frame 48 |  |  |
| 9804 | TOMM20 | -0.432 | 0.74 | -25.89 | 9.78 | 0.030 | translocase of outer mitochondrial membrane 20 homolog (yeast) | | |
| 80746 | TSEN2 | -0.432 | 0.74 | -25.86 | 3.53 | 0.048 | TSEN2 tRNA splicing endonuclease subunit | |  |
| 154 | ADRB2 | -0.429 | 0.74 | -25.71 | 8.23 | 0.038 | adrenoceptor beta 2, surface |  |  |
| 26586 | CKAP2 | -0.429 | 0.74 | -25.71 | 4.90 | 0.040 | cytoskeleton associated protein 2 |  |  |
| 89894 | TMEM116 | -0.428 | 0.74 | -25.66 | 4.06 | 0.024 | transmembrane protein 116 |  |  |
| 136895 | C7ORF31 | -0.427 | 0.74 | -25.61 | 4.71 | 0.003 | chromosome 7 open reading frame 31 |  |  |
| 8434 | RECK | -0.424 | 0.75 | -25.45 | 3.93 | 0.048 | reversion-inducing-cysteine-rich protein with kazal motifs | | |
| 6135 | RPL11 | -0.424 | 0.75 | -25.45 | 13.25 | 0.049 | ribosomal protein L11 |  |  |
| 900 | CCNG1 | -0.423 | 0.75 | -25.40 | 10.34 | 0.022 | cyclin G1 |  |  |
| 55024 | BANK1 | -0.423 | 0.75 | -25.39 | 7.20 | 0.047 | B-cell scaffold protein with ankyrin repeats 1 | |  |
| 55602 | CDKN2AIP | -0.419 | 0.75 | -25.22 | 6.75 | 0.020 | CDKN2A interacting protein |  |  |
| 94101 | ORMDL1 | -0.418 | 0.75 | -25.16 | 6.35 | 0.045 | ORM1-like 1 (S. cerevisiae) |  |  |
| 10208 | USPL1 | -0.416 | 0.75 | -25.03 | 7.27 | 0.028 | ubiquitin specific peptidase like 1 |  |  |
| 5128 | CDK17 | -0.413 | 0.75 | -24.90 | 5.19 | 0.047 | cyclin-dependent kinase 17 |  |  |
| 79657 | RPAP3 | -0.410 | 0.75 | -24.74 | 6.43 | 0.016 | RNA polymerase II associated protein 3 |  |  |
| 4154 | MBNL1 | -0.409 | 0.75 | -24.70 | 10.59 | 0.015 | muscleblind-like splicing regulator 1 |  |  |
| 256586 | LYSMD2 | -0.403 | 0.76 | -24.37 | 11.11 | 0.039 | LysM, putative peptidoglycan-binding, domain containing 2 | | |
| 5471 | PPAT | -0.402 | 0.76 | -24.31 | 2.88 | 0.044 | phosphoribosyl pyrophosphate amidotransferase | |  |
| 7336 | UBE2V2 | -0.401 | 0.76 | -24.26 | 7.82 | 0.023 | ubiquitin-conjugating enzyme E2 variant 2 |  |  |
| 84248 | FYTTD1 | -0.401 | 0.76 | -24.25 | 7.92 | 0.044 | forty-two-three domain containing 1 |  |  |
| 29945 | ANAPC4 | -0.399 | 0.76 | -24.18 | 7.67 | 0.031 | anaphase promoting complex subunit 4 |  |  |
| 387751 | GVINP1 | -0.397 | 0.76 | -24.04 | 7.85 | 0.047 | GTPase, very large interferon inducible pseudogene 1 | |  |
| 115426 | UHRF2 | -0.395 | 0.76 | -23.97 | 8.98 | 0.020 | ubiquitin-like with PHD and ring finger domains 2, E3 ubiquitin protein ligase | | |
| 51574 | LARP7 | -0.395 | 0.76 | -23.96 | 7.58 | 0.021 | La ribonucleoprotein domain family, member 7 | |  |
| 403313 | PPAPDC2 | -0.392 | 0.76 | -23.80 | 6.04 | 0.049 | phosphatidic acid phosphatase type 2 domain containing 2 | | |
| 8575 | PRKRA | -0.388 | 0.76 | -23.56 | 7.09 | 0.039 | protein kinase, interferon-inducible double stranded RNA dependent activator | | |
| 22890 | ZBTB1 | -0.387 | 0.76 | -23.51 | 6.13 | 0.028 | zinc finger and BTB domain containing 1 |  |  |
| 60526 | C2ORF43 | -0.384 | 0.77 | -23.37 | 3.99 | 0.041 | chromosome 2 open reading frame 43 |  |  |
| 113791 | PIK3IP1 | -0.370 | 0.77 | -22.62 | 9.10 | 0.047 | phosphoinositide-3-kinase interacting protein 1 | |  |
| 7163 | TPD52 | -0.370 | 0.77 | -22.61 | 5.44 | 0.022 | tumor protein D52 |  |  |
| 100505971 |  | -0.368 | 0.77 | -22.54 | 4.63 | 0.017 |  |  |  |
| 285331 | CCDC66 | -0.367 | 0.78 | -22.46 | 3.53 | 0.033 | coiled-coil domain containing 66 |  |  |
| 127018 | LYPLAL1 | -0.366 | 0.78 | -22.40 | 7.45 | 0.018 | lysophospholipase-like 1 |  |  |
| 29078 | NDUFAF4 | -0.364 | 0.78 | -22.33 | 3.99 | 0.045 | NADH dehydrogenase (ubiquinone) complex I, assembly factor 4 | | |
| 171586 | ABHD3 | -0.362 | 0.78 | -22.22 | 10.13 | 0.045 | abhydrolase domain containing 3 |  |  |
| 79017 | GGCT | -0.362 | 0.78 | -22.21 | 7.40 | 0.026 | gamma-glutamylcyclotransferase |  |  |
| 100506710 | LOC100506710 | -0.361 | 0.78 | -22.14 | 8.73 | 0.044 | endogenous Bornavirus-like nucleoprotein 2 pseudogene | | |
| 6342 | SCP2 | -0.361 | 0.78 | -22.12 | 10.10 | 0.047 | sterol carrier protein 2 |  |  |
| 132 | ADK | -0.358 | 0.78 | -21.96 | 4.09 | 0.031 | adenosine kinase |  |  |
| 285989 | ZNF789 | -0.355 | 0.78 | -21.80 | 4.21 | 0.045 | zinc finger protein 789 |  |  |
| 221294 | NT5DC1 | -0.354 | 0.78 | -21.75 | 5.44 | 0.048 | 5'-nucleotidase domain containing 1 |  |  |
| 2188 | FANCF | -0.350 | 0.78 | -21.57 | 5.45 | 0.027 | Fanconi anemia, complementation group F |  |  |
| 55299 | BRIX1 | -0.350 | 0.78 | -21.53 | 3.68 | 0.032 | BRX1, biogenesis of ribosomes, homolog (S. cerevisiae) | | |
| 100506969 |  | -0.350 | 0.78 | -21.52 | 6.10 | 0.031 |  |  |  |
| 203522 | DDX26B | -0.347 | 0.79 | -21.36 | 6.16 | 0.038 | DEAD/H (Asp-Glu-Ala-Asp/His) box polypeptide 26B | |  |
| 4673 | NAP1L1 | -0.346 | 0.79 | -21.34 | 8.54 | 0.048 | nucleosome assembly protein 1-like 1 |  |  |
| 7705 | ZNF146 | -0.346 | 0.79 | -21.30 | 4.48 | 0.020 | zinc finger protein 146 |  |  |
| 9666 | DZIP3 | -0.344 | 0.79 | -21.22 | 3.39 | 0.039 | DAZ interacting zinc finger protein 3 |  |  |
| 79886 | CAAP1 | -0.342 | 0.79 | -21.13 | 5.06 | 0.033 | caspase activity and apoptosis inhibitor 1 |  |  |
| 23347 | SMCHD1 | -0.340 | 0.79 | -21.00 | 10.86 | 0.041 | structural maintenance of chromosomes flexible hinge domain containing 1 | | |
| 11335 | CBX3 | -0.338 | 0.79 | -20.88 | 9.94 | 0.049 | chromobox homolog 3 |  |  |
| 29902 | FAM216A | -0.338 | 0.79 | -20.87 | 3.92 | 0.036 | family with sequence similarity 216, member A | |  |
| 5884 | RAD17 | -0.338 | 0.79 | -20.86 | 6.54 | 0.013 | RAD17 homolog (S. pombe) |  |  |
| 10473 | HMGN4 | -0.337 | 0.79 | -20.82 | 10.77 | 0.018 | high mobility group nucleosomal binding domain 4 | |  |
| 64924 | SLC30A5 | -0.336 | 0.79 | -20.78 | 6.38 | 0.046 | solute carrier family 30 (zinc transporter), member 5 | |  |
| 80218 | NAA50 | -0.333 | 0.79 | -20.62 | 7.80 | 0.032 | N(alpha)-acetyltransferase 50, NatE catalytic subunit | |  |
| 84992 | PIGY | -0.333 | 0.79 | -20.60 | 9.26 | 0.047 |  |  |  |
| 29883 | CNOT7 | -0.331 | 0.79 | -20.53 | 6.91 | 0.044 | CCR4-NOT transcription complex, subunit 7 | |  |
| 51012 | SLMO2 | -0.328 | 0.80 | -20.31 | 6.75 | 0.016 | slowmo homolog 2 (Drosophila) |  |  |
| 7763 | ZFAND5 | -0.327 | 0.80 | -20.28 | 11.48 | 0.013 | zinc finger, AN1-type domain 5 |  |  |
| 84329 | HVCN1 | -0.326 | 0.80 | -20.24 | 9.79 | 0.023 | hydrogen voltage-gated channel 1 |  |  |
| 5716 | PSMD10 | -0.326 | 0.80 | -20.23 | 7.76 | 0.045 | proteasome (prosome, macropain) 26S subunit, non-ATPase, 10 | | |
| 2971 | GTF3A | -0.325 | 0.80 | -20.16 | 11.76 | 0.040 | general transcription factor IIIA |  |  |
| 51562 | MBIP | -0.323 | 0.80 | -20.05 | 4.38 | 0.029 | MAP3K12 binding inhibitory protein 1 |  |  |
| 81853 | TMEM14B | -0.321 | 0.80 | -19.97 | 10.38 | 0.047 | transmembrane protein 14B |  |  |
| 139341 | FUNDC1 | -0.320 | 0.80 | -19.91 | 7.60 | 0.015 | FUN14 domain containing 1 |  |  |
| 100507399 | HCG8 | -0.320 | 0.80 | -19.89 | 2.50 | 0.047 | HLA complex group 8 |  |  |
| 7381 | UQCRB | -0.319 | 0.80 | -19.85 | 5.23 | 0.029 | ubiquinol-cytochrome c reductase binding protein | |  |
| 29767 | TMOD2 | -0.318 | 0.80 | -19.78 | 3.70 | 0.029 | tropomodulin 2 (neuronal) |  |  |
| 2339 | FNTA | -0.317 | 0.80 | -19.72 | 10.76 | 0.022 | farnesyltransferase, CAAX box, alpha |  |  |
| 23484 | LEPROTL1 | -0.312 | 0.81 | -19.47 | 10.20 | 0.013 | leptin receptor overlapping transcript-like 1 | |  |
| 23568 | ARL2BP | -0.312 | 0.81 | -19.45 | 6.70 | 0.033 | ADP-ribosylation factor-like 2 binding protein | |  |
| 8562 | DENR | -0.310 | 0.81 | -19.32 | 9.27 | 0.031 | density-regulated protein |  |  |
| 3796 | KIF2A | -0.309 | 0.81 | -19.28 | 8.44 | 0.028 | kinesin heavy chain member 2A |  |  |
| 51193 | ZNF639 | -0.304 | 0.81 | -19.01 | 4.72 | 0.037 | zinc finger protein 639 |  |  |
| 81688 | C6ORF62 | -0.302 | 0.81 | -18.87 | 10.66 | 0.018 | chromosome 6 open reading frame 62 |  |  |
| 84265 | POLR3GL | -0.296 | 0.81 | -18.55 | 9.05 | 0.015 | polymerase (RNA) III (DNA directed) polypeptide G (32kD)-like | | |
| 100506087 |  | -0.295 | 0.82 | -18.48 | 8.09 | 0.035 |  |  |  |
| 23635 | SSBP2 | -0.293 | 0.82 | -18.40 | 8.28 | 0.014 | single-stranded DNA binding protein 2 |  |  |
| 55330 | BLOC1S4 | -0.292 | 0.82 | -18.33 | 7.78 | 0.023 | biogenesis of lysosomal organelles complex-1, subunit 4, cappuccino | | |
| 7884 | SLBP | -0.292 | 0.82 | -18.32 | 9.73 | 0.015 | stem-loop binding protein |  |  |
| 25875 | LETMD1 | -0.291 | 0.82 | -18.29 | 8.69 | 0.047 | LETM1 domain containing 1 |  |  |
| 10541 | ANP32B | -0.291 | 0.82 | -18.26 | 12.38 | 0.030 | acidic (leucine-rich) nuclear phosphoprotein 32 family, member B | | |
| 55591 | VEZT | -0.286 | 0.82 | -17.99 | 3.11 | 0.026 | vezatin, adherens junctions transmembrane protein | |  |
| 5283 | PIGH | -0.285 | 0.82 | -17.91 | 7.66 | 0.039 | phosphatidylinositol glycan anchor biosynthesis, class H | | |
| 55082 | ARGLU1 | -0.276 | 0.83 | -17.39 | 8.10 | 0.047 | arginine and glutamate rich 1 |  |  |
| 689 | BTF3 | -0.273 | 0.83 | -17.25 | 13.45 | 0.023 | basic transcription factor 3 |  |  |
| 5734 | PTGER4 | -0.273 | 0.83 | -17.24 | 6.05 | 0.045 | prostaglandin E receptor 4 (subtype EP4) |  |  |
| 7852 | CXCR4 | -0.273 | 0.83 | -17.22 | 12.60 | 0.047 |  |  |  |
| 55278 | QRSL1 | -0.272 | 0.83 | -17.19 | 3.02 | 0.029 | glutaminyl-tRNA synthase (glutamine-hydrolyzing)-like 1 | | |
| 51669 | TMEM66 | -0.269 | 0.83 | -17.03 | 12.78 | 0.026 | transmembrane protein 66 |  |  |
| 55787 | TXLNG | -0.264 | 0.83 | -16.70 | 3.43 | 0.022 | taxilin gamma |  |  |
| 22889 | KIAA0907 | -0.263 | 0.83 | -16.64 | 9.40 | 0.030 | KIAA0907 |  |  |
| 64864 | RFX7 | -0.262 | 0.83 | -16.62 | 4.81 | 0.031 | regulatory factor X, 7 |  |  |
| 23029 | RBM34 | -0.260 | 0.83 | -16.50 | 4.78 | 0.010 | RNA binding motif protein 34 |  |  |
| 90411 | MCFD2 | -0.255 | 0.84 | -16.22 | 7.42 | 0.013 | multiple coagulation factor deficiency 2 |  |  |
| 90634 | N4BP2L1 | -0.254 | 0.84 | -16.17 | 4.88 | 0.048 | NEDD4 binding protein 2-like 1 |  |  |
| 11146 | GLMN | -0.254 | 0.84 | -16.12 | 3.05 | 0.047 | glomulin, FKBP associated protein |  |  |
| 79612 | NAA16 | -0.250 | 0.84 | -15.91 | 3.83 | 0.031 | N(alpha)-acetyltransferase 16, NatA auxiliary subunit | |  |
| 54680 | ZNHIT6 | -0.246 | 0.84 | -15.69 | 3.03 | 0.030 | zinc finger, HIT-type containing 6 |  |  |
| 9465 | AKAP7 | -0.246 | 0.84 | -15.68 | 5.76 | 0.044 | A kinase (PRKA) anchor protein 7 |  |  |
| 10286 | BCAS2 | -0.245 | 0.84 | -15.62 | 8.29 | 0.030 | breast carcinoma amplified sequence 2 |  |  |
| 100505641 | FGD5-AS1 | -0.243 | 0.85 | -15.50 | 9.99 | 0.045 | FGD5 antisense RNA 1 |  |  |
| 51696 | HECA | -0.240 | 0.85 | -15.34 | 11.28 | 0.030 | headcase homolog (Drosophila) |  |  |
| 84240 | ZCCHC9 | -0.237 | 0.85 | -15.16 | 7.96 | 0.046 | zinc finger, CCHC domain containing 9 |  |  |
| 4907 | NT5E | -0.233 | 0.85 | -14.93 | 2.80 | 0.030 | 5'-nucleotidase, ecto (CD73) |  |  |
| 80008 | TMEM156 | -0.227 | 0.85 | -14.58 | 4.57 | 0.020 | transmembrane protein 156 |  |  |
| 7109 | TRAPPC10 | -0.219 | 0.86 | -14.08 | 7.12 | 0.047 | trafficking protein particle complex 10 |  |  |
| 54842 | MFSD6 | -0.216 | 0.86 | -13.90 | 3.33 | 0.041 | major facilitator superfamily domain containing 6 | |  |
| 3189 | HNRNPH3 | -0.205 | 0.87 | -13.24 | 8.96 | 0.048 | heterogeneous nuclear ribonucleoprotein H3 (2H9) | |  |
| 100507463 | TAPSAR1 | -0.204 | 0.87 | -13.21 | 11.34 | 0.040 | TAP1 and PSMB8 antisense RNA 1 |  |  |
| 8634 | RTCA | -0.204 | 0.87 | -13.19 | 8.55 | 0.039 | RNA 3'-terminal phosphate cyclase |  |  |
| 54940 | OCIAD1 | -0.201 | 0.87 | -12.99 | 9.58 | 0.042 | OCIA domain containing 1 |  |  |
| 55573 | CDV3 | -0.198 | 0.87 | -12.85 | 8.50 | 0.028 | CDV3 homolog (mouse) |  |  |
| 84864 | MINA | -0.188 | 0.88 | -12.24 | 3.37 | 0.048 | MYC induced nuclear antigen |  |  |
| 10904 | BLCAP | -0.184 | 0.88 | -12.00 | 9.18 | 0.048 | bladder cancer associated protein |  |  |
| 55617 | TASP1 | -0.183 | 0.88 | -11.90 | 2.44 | 0.049 | taspase, threonine aspartase, 1 |  |  |
| 93621 | MRFAP1 | -0.178 | 0.88 | -11.59 | 13.17 | 0.018 | Morf4 family associated protein 1 |  |  |
| 9221 | NOLC1 | -0.177 | 0.88 | -11.53 | 4.45 | 0.031 | nucleolar and coiled-body phosphoprotein 1 | |  |
| 201229 | LYRM9 | -0.163 | 0.89 | -10.69 | 3.54 | 0.028 | LYR motif containing 9 |  |  |
| 54014 | BRWD1 | -0.161 | 0.89 | -10.53 | 3.25 | 0.047 | bromodomain and WD repeat domain containing 1 | |  |
| 5108 | PCM1 | -0.159 | 0.90 | -10.43 | 6.34 | 0.037 | pericentriolar material 1 |  |  |
| 10495 | ENOX2 | -0.083 | 0.94 | -5.62 | 2.92 | 0.050 | ecto-NOX disulfide-thiol exchanger 2 |  |  |
| 5191 | PEX7 | -0.068 | 0.95 | -4.63 | 2.48 | 0.042 | peroxisomal biogenesis factor 7 |  |  |
| 567 | B2M | -0.053 | 0.96 | -3.58 | 15.39 | 0.047 | beta-2-microglobulin |  |  |

**Table S3. Microarray and quantitative real time-PCR of the selected 28 genes.**

|  | **Forward Primer (5’ to 3’)** | **Reverse Primer (5’ to 3’)** | **RT-PCR** | | | | | **Microarray** | | | | |
| --- | --- | --- | --- | --- | --- | --- | --- | --- | --- | --- | --- | --- |
| **Gene** |  |  | **Fold Change**  **(Log_2_)** | **Fold**  **Change** | ***sPTB within 48 hours***  ***(median (25^th^, 75^th^ percentile))*** | ***No sPTB within 48 hours (median (25^th^, 75^th^ percentile)*** | ***LogXact***  ***p* Value** | **Fold**  **Change**  **(Log_2_)** | **Fold**  **Change** | ***sPTB within 48 hours***  ***(median (25^th^, 75^th^ percentile))*** | ***No sPTB within 48 hours (median (25^th^, 75^th^ percentile))*** | ***Limma* FDR** |
| *ANAPC10* | AGTTTCGGCAAGCAGCCAGAATA | TCCAACTGCTTGGGGTCAGCAC | -0.17 | 0.89 | 0.91 (0.70, 1.13) | 1.03 (0.82, 1.38) | 0.127 | -0.73 | 0.60 | 3.68 (3.31, 4.43) | 4.54 (3.66, 5.76) | 0.047 |
| *ANKRD46* | TGCTCGTTCGAGTCGCAGATCC | TTCGTTCAGTGGTCACTCAGCAGTT | -0.13 | 0.92 | 0.95 (0.78, 1.11) | 1.04 (0.74, 1.31) | 0.179 | -0.67 | 0.63 | 4.30 (3.89, 4.70) | 4.81 (4.17, 5.64) | 0.037 |
| *ATP9A* | GGCTCACAGCACGAGGCACAG | CATGTCGGCAGGGACCCGC | 0.85 | 1.80 | 1.22 (0.75, 3.05) | 0.68 (0.43, 1.36) | 0.010 | 0.88 | 1.84 | 7.86 (6.73, 8.92) | 6.71 (6.15, 7.78) | 0.013 |
| *C19orf59* | CAGCCAAGAATCAAGGTGCCCA | GGGACTTGGCTCGTGGGTCG | 0.67 | 1.59 | 1.35 (0.84, 2.31) | 0.84 (0.38, 1.25) | <0.001 | 0.70 | 1.63 | 12.85 (12.07, 13.38) | 12.18 (11.59, 12.64) | 0.005 |
| *CCDC104* | GAGCCCACAGTGCATTCCAGTGA | TTTTAACTTCTGAGGGTGGGTGTGC | -0.08 | 0.94 | 0.93 (0.77, 1.25) | 0.98 (0.75, 1.29) | 0.891 | -0.67 | 0.63 | 4.73 (3.95, 5.18) | 5.13 (4.39, 6.09) | 0.031 |
| *CCR1* | GTCCCTTGGAACCAGAGAGAAGCC | GGCACGGAGTTGCATCCCCATA | 0.67 | 1.59 | 1.34 (0.81, 1.85) | 0.85 (0.57, 1.13) | 0.003 | 0.60 | 1.52 | 11.57 (10.92, 12.12) | 10.97 (10.37, 11.35) | 0.022 |
| *CDK5RAP2* | ATTGCAGTCTCTCCGAGTGGAGCTG | TCTCCCGACGCCTCTCTCAGTCT | 0.44 | 1.35 | 1.19 (0.99, 1.40) | 0.88 (0.75, 1.05) | <0.001 | 0.77 | 1.70 | 8.73 (7.73, 10.10) | 8.12 (7.44, 8.80) | 0.017 |
| *CETN3* | TGGGCGTCTTGCTGCCTTGG | CCACTACAAGCTCACTTCTCAGAGC | -0.49 | 0.71 | 0.77 (0.59, 1.17) | 1.08 (0.70, 1.72) | 0.052 | -0.77 | 0.59 | 4.20 (3.01, 4.36) | 4.41 (3.98, 5.70) | 0.032 |
| *EMILIN2* | AACCCCAGCACCGGGGTCTT | GGCGTCTCTCTCGGGGGTGA | 0.60 | 1.52 | 1.36 (1.06, 1.72) | 0.89 (0.65, 1.16) | <0.001 | 0.61 | 1.53 | 7.29 (6.79, 7.84) | 6.83 (6.17, 7.20) | 0.015 |
| *EMR1* | TGCTCAACGGCCAGGTACGAGA | CCCGTCTTGGAAGCGGATGGC | 0.78 | 1.72 | 1.32 (0.75, 2.25) | 0.77 (0.65, 1.11) | 0.001 | 0.70 | 1.62 | 9.14 (8.64, 10.01) | 8.58 (7.89, 9.15) | 0.025 |
| *G0S2* | TGCCACTAAGGTCATTCCCGCCT | CCTTGCGCTTCTGGGCCATCA | 0.73 | 1.65 | 1.33 (0.96, 2.03) | 0.81 (0.60, 1.25) | <0.001 | 0.76 | 1.69 | 7.56 (7.10, 8.05) | 6.83 (6.26, 7.53) | 0.006 |
| *GALNT14* | AAGTGCAGACCCCTAAGCCTTCG | TCGTCACCAACGCGCCACTT | 0.80 | 1.74 | 1.30 (1.00, 2.15) | 0.75 (0.48, 1.16) | 0.001 | 0.88 | 1.84 | 9.04 (8.46, 9.58) | 8.27 (7.86, 8.66) | <0.001 |
| *GPR84* | CCACCAGAAAGGACTGCTCTTTGGG | GCTGTTCCACATGATAGAGGCTGAG | 0.96 | 1.94 | 1.36 (0.82, 2.38) | 0.70 (0.50, 1.28) | 0.001 | 0.99 | 1.99 | 8.00 (6.77, 8.78) | 6.78 (6.40, 7.64) | 0.007 |
| *IFT80* | TGGCTCCCTGGTTCACATCAGCA | TGGTTTGCTCCTTAACAAAGCGACA | -0.11 | 0.93 | 0.93 (0.73, 1.15) | 1.00 (0.78, 1.43) | 0.132 | -0.74 | 0.60 | 4.28 (3.39, 4.84) | 4.84 (4.01, 5.84) | 0.040 |
| *METTL18* | GTCCGGAGGAGGCGTTGTGAGG | TCCTCTTTCCAAGCAGCTCTGGGG | -0.41 | 0.75 | 0.79 (0.67, 1.11) | 1.05 (0.80, 1.48) | 0.018 | -0.91 | 0.53 | 3.46 (2.73, 3.79) | 4.20 (3.52, 5.00) | 0.002 |
| *MS4A1* | AGGCCTTGGAGACTCAGATCCTGA | TGTCAGTCTCTTCCCCACAGAATGG | -0.71 | 0.61 | 0.81 (0.56, 1.27) | 1.33 (0.75, 1.63) | 0.003 | -0.76 | 0.59 | 6.93 (6.09, 7.55) | 7.76 (6.97, 8.69) | 0.028 |
| *NLRP3* | CGCCCTCGGTGACTTCGGAA | CAGCAGCTGACCAACCAGAGCT | 0.52 | 1.43 | 1.19 (0.98, 1.89) | 0.83 (0.68, 1.09) | 0.001 | 0.65 | 1.57 | 4.83 (4.37, 5.39) | 4.42 (4.11, 4.75) | 0.015 |
| *OPLAH* | ATCAACACCGTGGCAGCGGG | ACAGGGCCCCCTTTGCGGTA | 0.57 | 1.48 | 1.20 (0.81, 2.01) | 0.81 (0.62, 1.27) | 0.003 | 0.93 | 1.91 | 6.62 (5.89, 7.90) | 5.82 (5.26, 6.69) | 0.003 |
| *PFKFB3* | CGCGAAGATGCCGTTGGAACTGA | GGCCCACAGGATCTGGGCAACGA | 0.80 | 1.74 | 1.44 (0.91, 1.89) | 0.82 (0.52, 1.19) | 0.001 | 0.68 | 1.61 | 11.52 (10.91, 12.32) | 10.95 (10.40, 11.40) | 0.012 |
| *PMS1* | GCGCTAGCAGGAAGCTGCTCTG | GCTCTTTTACAACACTGACCACCGA | -0.29 | 0.82 | 0.92 (0.79, 1.13) | 1.12 (0.82, 1.33) | 0.025 | -0.65 | 0.64 | 4.93 (4.40, 5.36) | 5.40 (4.62, 6.35) | 0.044 |
| *RAB20* | GGGGGCGCTGGGGGTTCTTA | GCGCCGCTCCATATACCGCT | 0.56 | 1.47 | 1.15 (0.81, 2.05) | 0.78 (0.62, 1.09) | 0.001 | 0.71 | 1.64 | 8.78 (8.35, 9.47) | 8.15 (7.65, 8.71) | 0.009 |
| *SCOC* | GCGCCTCAAGCGGAAGACCATT | ACTGCATCCATGTCAGCATTCATCA | -0.07 | 0.95 | 0.94 (0.73, 1.16) | 0.98 (0.76, 1.41) | 0.153 | -0.70 | 0.61 | 4.37 (3.80, 4.81) | 4.81 (4.31, 6.01) | 0.047 |
| *SOCS3* | CTGCGGGCTGGCGAAGGAAA | CCCAAGCCCTTCTCCCCGGA | 0.78 | 1.71 | 1.38 (0.81, 2.17) | 0.81 (0.53, 1.22) | 0.003 | 0.76 | 1.69 | 8.45 (7.51, 9.13) | 7.77 (7.14, 8.22) | 0.003 |
| *ST3GAL4* | TGCACATTGCCGGCTTTGGC | CCTGACCCCGCCATGGACTTG | 0.76 | 1.69 | 1.39 (0.91, 1.71) | 0.82 (0.63, 1.01) | <0.001 | 0.71 | 1.63 | 9.15 (8.41, 9.81) | 8.41 (7.71, 9.03) | 0.003 |
| *STAP1* | GCGAAATTCACCCTTGTTTTGCCG | GGGGAACTGACAGCTCTGTTACTGT | -0.60 | 0.66 | 0.81 (0.66, 1.19) | 1.23 (0.72, 1.61) | 0.007 | -0.76 | 0.59 | 4.10 (3.52, 4.52) | 4.78 (4.09, 5.50) | 0.007 |
| *TSPAN13* | ACCACTCGTGCTCGCCATGTG | GTCAGCCAAACACCCAGGATCTCTG | -0.03 | 0.98 | 0.97 (0.72, 1.35) | 1.00 (0.75, 1.18) | 0.766 | -0.61 | 0.66 | 6.32 (5.92, 6.80) | 7.09 (6.37, 7.55) | 0.022 |
| *ZDHHC19* | TCGGCCGACCGCACCTACAA | GCTTCAGCCATGTACTTGGGTCCC | 0.98 | 1.97 | 1.55 (0.84, 2.76) | 0.79 (0.26, 1.40) | 0.021 | 1.20 | 2.30 | 4.66 (3.95, 5.51) | 3.47 (3.19, 4.21) | <0.001 |
| *ZFAND1* | AGCATTGCCGGCAGCGAGATTTT | CAGGACAACCATGAGACTCCCTGCT | -0.29 | 0.82 | 0.87 (0.68, 1.17) | 1.06 (0.80, 1.35) | 0.058 | -0.77 | 0.59 | 5.35 (4.68, 6.31) | 6.18 (5.12, 6.99) | 0.040 |
| *SDHA* | TGGGAACAAGAGGGCATCTG | CCACCACTGCATCAAATTCATG | *(Housekeeping)* | | | | | *(Housekeeping)* | | | | |
| *TBP* | TGCACAGGAGCCAAGAGTGAA | CACATCACAGCTCCCCACCA | *(Housekeeping)* | | | | | *(Housekeeping)* | | | | |
| *YWHAZ* | ACTTTTGGTACATTGTGGCTTCAA | CCGCCAGGACAAACCAGTAT | *(Housekeeping)* | | | | | *(Housekeeping)* | | | | |
